# Neurogenesis in the embryonic and adult brain: same regulators, different roles

**DOI:** 10.3389/fncel.2014.00396

**Published:** 2014-11-27

**Authors:** Noelia Urbán, François Guillemot

**Affiliations:** Department of Molecular Neurobiology, MRC National Institute for Medical ResearchLondon, UK

**Keywords:** hippocampal neurogenesis, development of the hippocampus, regulation of adult neurogenesis, neural stem cell quiescence, niche signals in adult neurogenesis

## Abstract

Neurogenesis persists in adult mammals in specific brain areas, known as neurogenic niches. Adult neurogenesis is highly dynamic and is modulated by multiple physiological stimuli and pathological states. There is a strong interest in understanding how this process is regulated, particularly since active neuronal production has been demonstrated in both the hippocampus and the subventricular zone (SVZ) of adult humans. The molecular mechanisms that control neurogenesis have been extensively studied during embryonic development. Therefore, we have a broad knowledge of the intrinsic factors and extracellular signaling pathways driving proliferation and differentiation of embryonic neural precursors. Many of these factors also play important roles during adult neurogenesis, but essential differences exist in the biological responses of neural precursors in the embryonic and adult contexts. Because adult neural stem cells (NSCs) are normally found in a quiescent state, regulatory pathways can affect adult neurogenesis in ways that have no clear counterpart during embryogenesis. BMP signaling, for instance, regulates NSC behavior both during embryonic and adult neurogenesis. However, this pathway maintains stem cell proliferation in the embryo, while it promotes quiescence to prevent stem cell exhaustion in the adult brain. In this review, we will compare and contrast the functions of transcription factors (TFs) and other regulatory molecules in the embryonic brain and in adult neurogenic regions of the adult brain in the mouse, with a special focus on the hippocampal niche and on the regulation of the balance between quiescence and activation of adult NSCs in this region.

## Introduction

Neural stem cells (NSCs) in the embryonic and early postnatal murine brain generate neurons and glia, including astrocytes and oligodendrocytes. The transition of proliferative and multipotent NSCs to fully differentiated neurons and glia is called neurogenesis and gliogenesis, respectively. Neurons are generated from early embryonic development until early postnatal stages, with only a few neurogenic zones remaining active in the adult (Götz and Huttner, 2005; Ming and Song, 2011; Paridaen and Huttner, 2014). In contrast, gliogenesis starts during late embryogenesis and continues in postnatal stages, with low but widespread production of both astrocytes and oligodendrocytes also occurring throughout the adult brain (Rowitch and Kriegstein, 2010; Gallo and Deneen, 2014; Guérout et al., 2014). The main neurogenic regions in the adult murine brain are the subependymal zone of the lateral ventricles, also called ventricular-subventricular Zone (V-SVZ) and the subgranular zone (SGZ) of the dentate gyrus (DG) in the hippocampus (Altman and Das, 1965; Doetsch et al., 1999; Ming and Song, 2011; Fuentealba et al., 2012). Both of these neurogenic regions have been shown to also be active in the adult human brain, with the V-SVZ thought to contribute new neurons to the striatum (whereas it produces neurons migrating to the olfactory bulb in mice) and the SGZ contributing neurons to the DG (Eriksson et al., 1998; Spalding et al., 2013; Ernst et al., 2014). The addition of new neurons to the complex circuitry of the adult brain is the focus of intensive research, which is uncovering crucial functions for the newly generated neurons in memory and behavior (Deng et al., 2010). In particular, the integration of adult-born granule cells to the hippocampus circuitry confers an extra degree of plasticity that is crucial for the acquisition of certain types of contextual memory (Jessberger et al., 2009; Sahay et al., 2011). Although adult neurogenesis is an ancient trait, with widespread neurogenesis occurring, for instance, in 16 different adult brain areas of zebrafish, the appearance of the DG as a structural and functional unit seems exclusive to mammals (Treves et al., 2008; Grandel and Brand, 2013). This fact, amongst others, has prompted the idea that hippocampal neurogenesis might be a newly evolved trait in some species, including humans, aimed to enhance adaptation to a continuously changing environment (Kempermann, 2012).

Significant advances have been made in our understanding of the regulation of mouse adult hippocampal neurogenesis in the last few years. Thus, our focus for the rest of the review will be on the mouse model of neurogenesis. The coordinated action of multiple signals acting on embryonic NSCs gives rise to the vast diversity of neuronal and glial populations that populate the mature brain. Embryonic neurogenesis is, thus, tightly linked to cell fate specification. In adult neurogenic regions, however, stem cells are tightly restricted to the generation of one (granule neurons of the DG) or a few types of neurons (granule neurons and periglomerular neurons in the V-SVZ) (Zhao et al., 2008; Ming and Song, 2011). Therefore signals and factors that specify subtype identities during development can control more subtle aspects of adult stem cell behavior.

In recent years, it has become evident that, at the single cell level, stem cells in the embryonic and the adult brain are not as versatile as previously thought. Instead of their classically attributed multipotency, they appear to be already committed to the generation of specific types of neural cells (Taverna et al., 2014). The causes and functions of the emerging heterogeneity of adult NSCs are among the most exciting questions remaining to be addressed in the field (DeCarolis et al., 2013; Encinas et al., 2013; Giachino et al., 2014b). In the case of the murine V-SVZ, different populations of adult NSCs, also called type-B cells, co-exist and give rise to distinct types of periglomerular cells and granule cells in the olfactory bulb. Different adult NSCs are characterized by the differential expression of specific transcription factors (TFs), including Nkx2.1, Pax6, Gsx2 and Nkx6.2, which also pattern the different domains of the embryonic telencephalon (Merkle et al., 2007; Brill et al., 2008; López-Juárez et al., 2013; Merkle et al., 2014). The distinct adult NSC populations are located in different regions along the V-SVZ and their distinct properties are acquired during development (Obernier et al., 2014). Despite the spatial separation of these stem cell populations, all their progeny follow the same long migratory path, the rostral migratory stream (RMS), towards their final destination in the olfactory bulb. In the hippocampus, adult NSCs, also called type-I cells or radial glial-like cells, generate exclusively granule neurons in the DG. The migration of granule neurons is very limited, as they settle, differentiate and integrate into the hippocampal circuitry in the granule cell layer (GCL) located just above the NSC from which they originated in the SGZ. While they appear uniform, adult NSCs in the DG respond to diverse and complex signals, raising the possibility that they are functionally heterogeneous.

Despite their many differences, adult NSCs in the two adult neurogenic niches share several key characteristics. Neural stem cells in both V-SVZ and SGZ, like radial glial stem cells in the embryo, express the molecules GFAP, Nestin and Sox2 and they directly contact blood vessels. Both NSC populations share a restricted potential, as just discussed, with each generating a unique neuronal subtype and one type of glia: in the V-SVZ they generate neurons and oligodendrocytes, while in the SGZ they generate neurons and astrocytes. Perhaps the two characteristics that distinguish adult NSCs most clearly from their embryonic counterparts are the acquisition of quiescence and their situation in a complex and stable cellular niche. While one of the main features of embryonic NSCs is their high proliferative rate, the opposite is true for adult NSCs, which remain for long periods out of the cell cycle, in G0. This is a characteristic that adult NSCs share with many stem cells in other mature tissues and one that is crucial to maintain tissue homeostasis and avoid stem cell exhaustion (Orford and Scadden, 2008; Simons and Clevers, 2011). The existence of adult neurogenic niches (complex cellular microenvironments surrounding adult NSCs) is also a characteristic shared with other tissues (Fuchs et al., 2004; Kuang et al., 2008; Mirzadeh et al., 2008; Ming and Song, 2011; Fuentealba et al., 2012; Goldstein and Horsley, 2012). The niche is comprised of diverse cell types and structures, such as astrocytes, neurons, axon projections and blood vessels, and one of its main functions is to create an appropriate environment that keeps the majority of stem cells quiescent and undifferentiated (Morrison and Spradling, 2008). The niche also provides a great variety of signals that modulate the behavior of adult stem cells and adjust the production of new cells to the needs of the tissue (Fuchs et al., 2004; Blank et al., 2008; Faigle and Song, 2013).

In this review we will consider the role of different extrinsic and intrinsic factors on NSCs, comparing their action during adult hippocampal neurogenesis with that reported in the embryonic brain or in the adult V-SVZ.

## Embryonic and adult origin of granule cells

**Figure 1:**
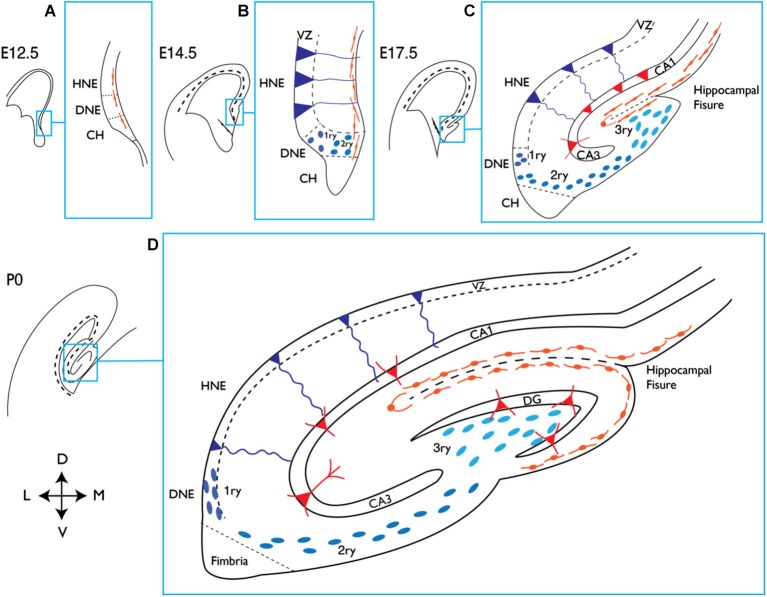
**Development of the mouse hippocampus**. Schematic representation of the dorsal telencephalon at different embryonic (E) stages and at birth (P0). The indicated area in each picture corresponds to the hippocampal region and is magnified on its right handside (blue squares). **(A)** At E12.5 the presumptive DNE is located between the HNE and the CH, which produces Cajal-Retzius cells (orange), shown lining the pial side of the cortex. **(B)** At E14.5 dentate precursors of the primary matrix (dark blue circles) are located in the VZ, and precursor cells start to migrate towards the pial side of the cortex forming the secondary matrix. In the VZ of the HNE, radial glial precursors (depicted in dark blue and triangular body shape) will give rise to hippocampal neurons. **(C)** At E17.5 the hippocampal fissure is formed and dentate precursor cells migrate to and accumulate there, forming the tertiary matrix (light blue). Cajal-Retzius cells are also present and follow the hippocampal fissure. At this stage the glial scaffold (not shown) extends from the CH to the hippocampal fissure and pial surface, directing the migration of dentate precursor cells. From the HNE, hippocampal neurons (red triangles) are born and migrate along radial glial cells towards their location in the hippocampal fields (CA1 and CA3 are shown). **(D)** At birth the blades of the DG start to form. Granule neurons in the DG (red triangles) appear first in the upper blade, below the hippocampal fissure. The continuous migration of Cajal-Retzius cells reaches the pial side and promotes the formation of the lower blade of the DG. Precursor cells in the primary and secondary matrix will soon disappear, but cells in the tertiary matrix continue actively dividing and producing granule neurons through postnatal DG development. HNE, hippocampal neuroepithelium; DNE, dentate neuroepithelium; CH, cortical hem; VZ, ventricular zone; 1ry, primary matrix; 2ry, secondary matrix; 3ry, tertiary matrix; DG, dentate gyrus; D, dorsal; M, medial; V, ventral; L, lateral.

From a developmental point of view, the generation of the DG is unique. While the V-SVZ is seen as a continuation of the embryonic ventricular zone (VZ) of the telencephalon, the formation of the DG involves the generation of a dedicated progenitor cell source away from the VZ and in close proximity to the pial surface. This additional proliferative zone remains active during postnatal stages and eventually becomes the SGZ, which is the site of adult hippocampal neurogenesis (Figure 1; Bayer, 1980a,b; Altman and Bayer, 1990; Pleasure et al., 2000; Khalaf-Nazzal and Francis, 2013; Sugiyama et al., 2013).

**Figure 2:**
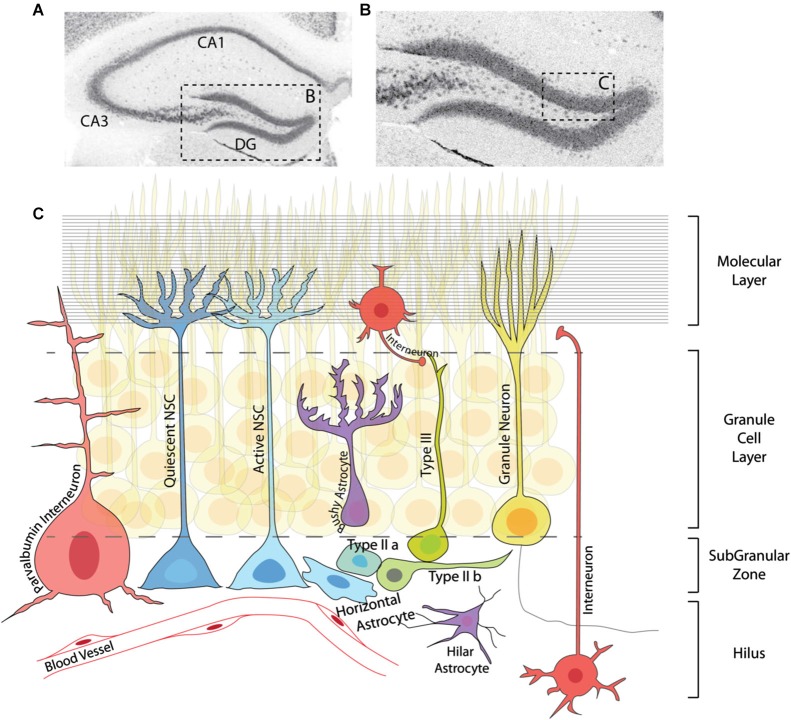
**Adult neurogenesis in the dentate gyrus. (A)** Immunohistochemistry for the neuronal marker NeuN showing the structure of the adult hippocampus. **(B)** Magnification of the DG region in **(A)**. **(C)** Graphic representation of the area marked in **(B)** depicting the neurogenic lineage and several elements of the DG niche. The neurogenic lineage consists of quiescent and active NSCs (including horizontal astrocytes), IPCs (typeIIa, typeIIb), neuroblasts (typeIII) and granule neurons. Neural stem cells and IPCs reside in the SGZ, while neuroblasts and neurons are found in the granule cell layer. Several types of interneurons (red) and astrocytes (purple) are located in different regions of the DG, and together with granule neurons are essential parts of the adult hippocampal niche. Blood vessels throughout the DG and axonal projections in the molecular layer (horizontal lines) also contribute to the regulation of adult neurogenesis at different steps of the lineage.

The DG originates from the dentate neuroepithelium (DNE), also called primary matrix, a part of the VZ of the medial pallium that is in direct contact with the cortical hem (CH) and becomes clearly distinguishable from embryonic day 14.5 (E14.5; Figures 1A,B). At late gestational stages, progenitor cells migrate out of the DNE towards the pial side of the medial cortex in a process that depends on hem-derived Cajal-Retzius cells (Rickmann et al., 1987; Del Río et al., 1997). These progenitors, which consist of a heterogeneous mixture of stem cells and neuronal precursors at different stages of differentiation, migrate away from the VZ towards the hippocampal fissure, constituting a new migratory progenitor population called the secondary matrix (Figures 1B,C). At the same time, a glial scaffold develops and bridges the fimbria to the pial side of the cortex and the hippocampal fissure. Both the glial scaffold and Cajal-Retzius cells remain present throughout DG development and have essential roles in the migration and organization of dentate precursor cells and granule neurons. Neural progenitors reach the hippocampal fissure, where they accumulate and form yet another hub of proliferating cells called the tertiary matrix (Figures 1C,D). Granule cells generated during DG development from precursors of all three matrices, form the GCL. Its characteristic shape of two blades is dictated by the Cajal-Retzius cells surrounding the hippocampal fissure and the pial surface (Figure 1D). By early postnatal stages, the tertiary matrix becomes the only source of dentate progenitors and granule cells. During the second postnatal week, proliferation in the DG becomes even more restricted and is eventually confined to the SGZ, where NSCs reside throughout adulthood (Figure 2).

One of the most interesting and still partially unresolved aspects of DG development is the question of the origin of the adult NSCs. The classical view is that they originate from the whole length of the DNE, from where GFAP-expressing cells migrate towards the SGZ, side by side with differentiating neuronal precursors (Seri et al., 2004; Li and Pleasure, 2005). These GFAP^+^ progenitors can give rise to granule neurons from early stages of DG development through adulthood (Seki et al., 2014). However, genetic cell lineage tracing of Sonic Hedgehog (SHH)-responsive cells has recently challenged this view, revealing that adult NSCs are induced at peri-natal stages in a restricted region next to the ventral-most side of the hippocampus, in close proximity to the lateral ventricle. Adult NSCs are induced there by SHH secreted from the amygdala and then migrate to populate all regions of the DG (Li et al., 2013). The separate origin of embryonic and adult NSCs in the DG could have important implications for the function and regulation of adult hippocampal neurogenesis.

## Regulation of adult neurogenesis

The late maturation of the hippocampus, which spans late embryonic and early postnatal stages, means that the process of DG formation and the appearance of NSCs with adult characteristics are overlapping processes. It can therefore be difficult to distinguish between developmental and adult cues regulating hippocampal neurogenesis. However, several physiological and pathological situations, such as physical exercise, task learning, an enriched environment and seizures, have been shown to stimulate neurogenesis specifically in the adult DG (Rolando and Taylor, 2014). Although no direct link has been clearly established between those external stimuli and signaling pathways, numerous extracellular signaling molecules, including Bone Morphogenetic Proteins (BMPs), Notch, GABA, WNT, insulin growth factors (IGFs) and SHH, have been shown to regulate the rate of neurogenesis in the adult DG (Ming and Song, 2011; Faigle and Song, 2013). However, due to limitations of *in vivo* studies, little is known about the mechanisms by which these signals exert their effects. In the adult DG, NSCs generate granule cells via a well characterized cell lineage that includes a succession of transit amplifying or intermediate progenitor cells (IPCs), characterized by rapid divisions and the expression of a series of neurogenic TFs (Figure 2; Hsieh, 2012). Extrinsic stimuli can affect the proliferation and survival of NSCs but also of IPCs (typeIIa and typeIIb) or differentiating neuroblasts (typeIII) further along the lineage (Figure 2). The selective death of IPCs, for instance, is a major mechanism of regulation of neurogenesis in the DG, with as many as two thirds of these cells being actively eliminated by microglia (Sierra et al., 2010, 2014). Therefore, in order to understand the effects of signaling pathways and intrinsic factors on neurogenesis, it is crucial to determine the stages in the adult neurogenic lineage at which they act, and the cellular processes they regulate. In fact, one of the main difficulties faced by the adult neurogenesis field concerns the scarcity of markers for adult NSCs, which are often shared by other cell types (for instance, GFAP marks subpopulations of astrocytes and Nestin is expressed by early intermediate progenitors). This problem is only more evident in the case of distinguishing quiescent from activated adult NSCs, in which case there is an absolute lack of specific markers apart from the use of cell cycle genes. This issue has been partly addressed in a recent report in which an unbiased approach was used to identify genes differentially expressed by activated and quiescent adult NSCs isolated from the V-SVZ (Codega et al., 2014). This work demonstrates that the quiescent state is a much more complex state than simply the lack of proliferation markers, as the list of differentially expressed genes is enriched in genes related to very diverse cellular processes, such as lipids metabolism, signaling or adhesion. This quiescence signature is shared by adult quiescent stem cells from other organs, such as the blood, muscle or intestine (Cheung and Rando, 2013; Codega et al., 2014). It is thus likely that many of the general characteristics of quiescent stem cells will be shared between DG and SVZ, although no studies on the expression profile of adult DG NSCs have been performed to date.

Ageing of the brain is marked by a major decrease in the number of new neurons generated in the DG. This decrease has been attributed both to a reduction of the NSC pool and to an increased state of quiescence of the remaining stem cells (Lugert et al., 2010; Encinas et al., 2011; Jaskelioff et al., 2011; Seib et al., 2013). The possibility to increase neurogenesis in ageing mice by activating the quiescent stem cell pool is currently the focus of intensive research. In this regard, it was recently shown that systemic factors from young animals can re-activate neurogenesis in aged mice (Katsimpardi et al., 2014). However, disruption of quiescence signals can lead to a short-lived increase in neurogenesis, followed by a sharp decrease caused by a loss of quiescent NSCs (Ehm et al., 2010; Mira et al., 2010; Song et al., 2012). Assessing precisely how factors and signals affect stem cell behavior will be vital to understand their long-term effects on adult neurogenesis. Lineage tracing and particularly clonal analysis of NSCs in the DG have begun to provide evidence of the great diversity of responses of adult NSCs to stimuli, which can affect both their proliferation and differentiation potentials (Bonaguidi et al., 2011; Dranovsky et al., 2011; Song et al., 2012).

## Cortical hem signals: BMP and WNT

The formation of the hippocampus starts in the mouse around E14 in response to the signals emanating from the CH, a dorso-medial telencephalic structure that acts as an organizer for the hippocampus and the choroid plexus (Grove et al., 1998; Mangale et al., 2008). The hem is characterized by the active secretion of BMP and WNT molecules and the lack of expression of the TF Lhx2. The crucial role of the hem in the formation of the hippocampus is demonstrated by both loss and gain of function studies. When the hem fails to form, the hippocampus does not develop properly (Yoshida et al., 2006), while in *Lhx2* mutant chimeric embryos, the hem-like *Lhx2*-negative tissue is able to induce hippocampal gene expression in ectopic areas of the dorsal telencephalon (Mangale et al., 2008). Bone Morphogenetic Protein and WNT signals have critical functions in hippocampal development, particularly to promote the proliferation of neural precursors (Furuta et al., 1997; Galceran et al., 1999; Lee et al., 2000; Caronia et al., 2010). In the adult, both signals continue to be extremely important for NSCs maintenance and differentiation, but their effects are very different, as discussed below.

Multiple **BMP**s (BMP4, BMP5, BMP6 and BMP7) are produced early on by the telencephalic roof plate and later by the CH (Furuta et al., 1997; Grove et al., 1998; Hébert et al., 2002). The complete loss of BMP signaling results in the absence of medio-dorsal structures, including the choroid plexus and the CH, resulting in the absence of the hippocampus (Cheng et al., 2006; Fernandes et al., 2007). Once the CH is formed however, BMPs do not seem to be necessary any longer for specification of hippocampal cell identities (Hébert et al., 2002). The effects that BMPs exert on neural precursors are very diverse, possibly due to the different activities of type 1 BMP receptors (BMPR-I), including BMPR-Ia, which promotes proliferation in the embryonic telencephalon, and BMPR-Ib, which induces cell cycle arrest and differentiation (Panchision et al., 2001).

In the adult DG, BMPs are indispensable for the maintenance of the quiescent state of NSCs (Mira et al., 2010). Bone Morphogenetic Proteins are chronically secreted by granule neurons and by NSCs themselves, and several BMP inhibitors, including Noggin and Chordin, are also present in the hippocampal niche (Scott et al., 2000; Fan et al., 2003; Bonaguidi et al., 2005, 2008). Loss of BMP signaling by deletion of the BMPR-Ia receptor subunit leads to an over-activation of adult NSCs that ultimately depletes their population (Mira et al., 2010). Bone Morphogenetic Proteins can also induce quiescence in NSCs in culture, providing a useful model to study in depth the molecular pathways regulating stem cell behavior (Mira et al., 2010; Sun et al., 2011; Martynoga et al., 2013). In this *in vitro* system, proliferating NSCs treated with BMP4 in combination with FGF2 rapidly exit the cell cycle and can be maintained for several weeks in a state of reversible cell cycle arrest, in which cells retain their proliferation and neurogenic potentials (Martynoga et al., 2013). Bone Morphogenetic Proteins can also promote the expression of astrocytic genes *in vitro*, raising the possibility that they induce some of the astroglial features of adult NSCs (Gross et al., 1996; Sun et al., 2011). Bone Morphogenetic Proteins are necessary not only for the quiescence of NSCs, but also for the differentiation and maturation of granule cells (Bond et al., 2014). This dual role might be explained by the differential expression of the BMPR-I receptors. Neural stem cells in the adult DG express BMPR-Ia, which is downregulated in IPCs, while neuroblasts and neurons express BMPR-Ib (Mira et al., 2010). Therefore, both NSCs and neuroblasts receive BMP signals, which they interpret as quiescence and differentiation cues, respectively.

The effects of BMP signaling on adult neurogenesis in the V-SVZ are less well understood (Lim et al., 2000; Colak et al., 2008). There is currently no clear evidence supporting a role for BMPs in maintaining the quiescence of V-SVZ stem cells, and the BMP inhibitor Noggin does not affect the behavior of V-SVZ-derived stem cells while it promotes the expansion of DG-derived stem cells *in vitro* (Bonaguidi et al., 2008).

The embryonic CH produces several **WNT** proteins, including WNT2a, WNT2b, WNT3a and WNT5a, which are instrumental for its role as hippocampal organizer. For instance, disruption of WNT3a prevents the formation of the hippocampus, thus establishing the absolute requirement for WNTs in hippocampal development (Lee et al., 2000). Disruption of Lef1, the main downstream effector of canonical WNT signaling, or of the WNT receptor Lrp6, also causes severe hippocampal defects (Galceran et al., 1999; Yoshida et al., 2006). Moreover, ectopic expression of Lef1 is sufficient to specify particular hippocampal domains, thus demonstrating that WNT activation is sufficient to confer hippocampal identity (Machon et al., 2007). WNTs are also involved in the formation of the glial scaffold, which is required during DG development for the migration of neural precursor cells from the VZ in the medial pallium to their final location in the hippocampus (Zhou et al., 2004).

WNT signaling also plays a role in postnatal and adult neurogenesis (Zhang et al., 2011; Ortiz-Matamoros et al., 2013; Varela-Nallar and Inestrosa, 2013). WNTs are secreted by astrocytes and by stem cells and are therefore thought to act both in a paracrine and an autocrine manner (Lie et al., 2005; Qu et al., 2010; Okamoto et al., 2011). WNTs can directly induce neurogenic genes such as Neurog2, NeuroD1 and Prox1 in intermediate progenitors, and they have a well-established role in synapse formation and the maturation of adult-born neurons (Kuwabara et al., 2009). Inhibition of canonical WNT signaling in the DG *in vivo* has adverse effects on the performance of mice in DG-dependant behavioral tests, suggesting that this pathway regulates adult hippocampal neurogenesis. In addition to promoting neuronal differentiation and maturation, several *in vitro* studies have shown that canonical WNT signaling also affects the proliferation of hippocampal progenitors (Lie et al., 2005; Varela-Nallar and Inestrosa, 2013). Moreover, a recent genetic analysis of the WNT inhibitor secreted frizzled related protein 3 (SFRP3) in the DG *in vivo*, also provides support for a role of WNT signaling in adult NSC activity. Loss of SFRP3, which is normally tonically secreted by granule cells, resulted in excessive proliferation of NSCs and the loss of the quiescent stem cell pool (Jang et al., 2013). Neuronal activity was shown to decrease the production of SFRP3 by granule cells, thus establishing a molecular link between neuronal activity and neurogenesis in the DG. In another study, deletion of the WNT inhibitor Dickkopf-related protein 1 (Dkk1) from granule neurons was sufficient to restore hippocampal neurogenesis in old mice (Seib et al., 2013). This finding demonstrates that the decline in neurogenesis in aged mice results, at least in part, from the increased quiescence of NSCs, and not only from the diminution of the stem cell pool, and suggests that this decline can be reversed.

Because BMP and WNT signals act at multiple steps of the neurogenic lineage during both embryonic and adult neurogenesis, it is difficult to assess their specific contribution to the regulation of NSCs. Nevertheless, current evidence indicates that WNT signaling maintains its pro-proliferative function from embryonic to adult NSCs and that modulation of its activity by WNT antagonists secreted by surrounding granule neurons is a critical aspect of the regulation of NSCs by the hippocampal niche. In contrast, BMP signaling through BMPR-Ia changes drastically its function from embryonic neural precursors, where it is mitogenic, to adult DG stem cells where it is a potent inducer of quiescence.

## Notch signaling

The functions of Notch signaling during embryonic brain development have been extensively reviewed elsewhere (Kageyama et al., 2008; Imayoshi and Kageyama, 2011). During development of the hippocampus, Notch does not seem to be involved in neural precursor specification or differentiation, but rather in broader decisions, including the regulation of neural lineage commitment, the tempo of neuronal and glial generation and the maintenance of stem cells. Notch receptors and ligands are broadly expressed during all stages of development of the hippocampus (Pleasure et al., 2000). Loss of the essential Notch signaling component RBPJk in the developing brain results in proliferation defects and premature differentiation of embryonic NSCs (Imayoshi et al., 2010). Similarly, loss of RBPJk or of the Notch ligand Jagged1 during hippocampal development leads to defects in proliferation and stem cell maintenance, although the formation of the DG is not prevented (Breunig et al., 2007; Lavado and Oliver, 2014). Therefore, the main function of the Notch pathway in embryonic NSCs is to maintain their proliferative and undifferentiated state.

High levels of Notch signaling, assessed by the expression of the Notch targets Hes1 and Hes5, are present in NSCs in the adult DG (Imayoshi et al., 2010; Lugert et al., 2010). The main Notch ligand is Jagged1, which is expressed both by the niche astrocytes and by IPCs, although other ligands, such as Dll1, Dll3 or Dll4, and other cellular sources, such as endothelial cells and NSCs themselves, could also contribute to maintaining Notch activity in NSCs (Stump et al., 2002; Lavado and Oliver, 2014). The interaction between Jagged1 on IPCs and Notch receptors on NSCs has been proposed to function as a feedback mechanism maintaining NSC quiescence. Elimination of the IPC population leads to an initial increase in NSC activation and IPC production followed by the exhaustion of the stem cell pool (Lavado et al., 2010; Hodge et al., 2012). The same sequence of events also takes place when Notch signaling is ablated cell autonomously in NSCs by deletion of the Notch1 receptor or of RBPJk (Ables et al., 2010; Ehm et al., 2010; Lavado and Oliver, 2014).

The effects of Notch on adult neurogenesis are context-dependent, as also reported in other adult stem cell niches (Mourikis and Tajbakhsh, 2014). In particular, signaling through Notch1 appears to have different functions in the DG and the V-SVZ. Loss of Notch1 in the DG leads to a significant decrease in the number of NSCs, while Notch1 deletion in the V-SVZ impairs the proliferation of NSCs without affecting their total number (Ables et al., 2010; Basak et al., 2012). The role of Notch signaling in adult neurogenesis has also been studied in zebrafish, where, as in the mammalian hippocampus, it is essential to keep stem cells quiescent (Chapouton et al., 2010, 2011). In zebrafish, different Notch receptors appear to operate at different steps of neurogenesis and to regulate different properties of stem cells, with Notch3 activity maintaining quiescence and Notch1 being required to prevent premature stem cell differentiation (Alunni et al., 2013). In the mammalian brain as well, divergent functions of the different Notch receptors might contribute to the heterogeneity of the responses of adult progenitor cells to Notch ligands (Shimizu et al., 2002; Giachino and Taylor, 2014). It is worth noting that perturbing Notch signaling has both cell autonomous and cell non-autonomous consequences, which complicates the dissection of its specific roles in adult neurogenesis.

A tantalizing hypothesis for the mechanism underlying Notch function in stem cell quiescence comes from embryonic data showing that the levels of Hes proteins and proneural bHLHs oscillate in neural precursor cells (Imayoshi et al., 2013). Hes proteins are bHLH TFs that are induced by Notch activity and act as potent repressors of gene expression, and proneural bHLH genes are amongst their main targets (Imayoshi and Kageyama, 2014). Hes transcripts and proteins oscillate with a frequency of 2–3 h, because Hes proteins repress their own transcription and because this repression is only transient due to their short half-lives (Shimojo et al., 2008; Imayoshi et al., 2013). The oscillation of Hes proteins drives in opposite phase the oscillation of their targets, including the proneural proteins Neurog2 and Ascl1 (Shimojo et al., 2008; Imayoshi et al., 2013). Ascl1 has been shown to have two opposing roles in embryonic neurogenesis, promoting progenitor proliferation and driving their cell cycle exit and differentiation (Castro et al., 2011). Interestingly, the oscillating expression of Ascl1 promotes the proliferation of neural progenitors, while its stable expression instead drives differentiation (Imayoshi et al., 2013). The mechanisms that convert different Ascl1 dynamics into the activation of different gene expression programmes, promoting proliferation and differentiation, respectively, remain unknown. Whether Hes and Ascl1 proteins also oscillate in adult progenitors has not yet been established. Adult NSCs express high levels of Hes proteins, but an initial reduction in the amount of Notch signaling might initiate their oscillatory expression, which would thus trigger the oscillation of Ascl1 expression and the proliferation of NSCs. Subsequently, a complete loss of Notch activity in IPCs would stabilize Ascl1 expression and promote neuronal differentiation. Several observations support such a scenario. Ascl1 expression is indeed increased upon loss of RPBJk in NSCs, showing that also in the adult DG, Notch signaling suppresses its expression (Andersen et al., 2014). Differences in the intensity of Notch signaling have been singled out as possible causes of the heterogeneity in adult NSC behavior (Giachino and Taylor, 2014). Moreover, Ascl1 expression in adult NSCs is excluded from quiescent NSCs and confined to about a third of activated NSCs, suggesting that Ascl1 has indeed a dynamic expression in proliferating NSCs (Andersen et al., 2014). Further analysis will determine the importance of the interactions between the Notch-Hes pathways and Ascl1 in regulating the transitions between quiescent and activated NSCs and between NSCs and IPCs.

## Neurogenic TFs: Neurog2, Tbr2 and Prox1

**Neurog2** is a bHLH TF with proneural activity in the embryonic brain, as it promotes the neuronal commitment of multipotent stem cells and induces the expression of other genes involved in neuronal differentiation, such as the NeuroD family of TFs (Seo et al., 2007; Wilkinson et al., 2013). Neurog2 also has a prime role in the specification of glutamatergic neurons in the embryonic brain (Schuurmans et al., 2004; Berninger et al., 2007; Wilkinson et al., 2013). During DG development, precursor cells in all proliferative matrices express Neurog2 (Pleasure et al., 2000; Galichet et al., 2008). Neurog2 has an essential role in the formation of the DG, as shown by the analysis of *Neurog2* null mutant mice, that present at birth a severely atrophic DG, with an upper blade greatly reduced in size and a lower blade missing (Galichet et al., 2008). Progenitors in the *Neurog2* mutant DG present both proliferation and differentiation defects, and although *Ascl1* is also expressed by progenitor cells during DG morphogenesis, it does not compensate for the loss of *Neurog2*, in contrast with what is observed in the embryonic telencephalon (Nieto et al., 2001; Galichet et al., 2008). In addition, the glial scaffold is disorganized in the *Neurog2* mutant DG, suggesting that progenitor migration is also disrupted (Galichet et al., 2008). The disorganization of the glial scaffold and atrophy of the DG are reminiscent of the phenotypes observed in WNT mutant embryos, and since *Neurog2* expression has been reported to be regulated by WNT signaling in the embryonic brain, Neurog2 might act as an effector of WNT signaling during DG formation (Hirabayashi et al., 2004; Zhou et al., 2004; Galichet et al., 2008).

In the adult V-SVZ, Neurog2 is expressed by a subset of IPCs that differentiate into glutamatergic interneurons (Brill et al., 2009). Neurog2 is also expressed transiently by a subset of Tbr2 positive IPCs in the DG (Hodge et al., 2008). NeuroD1, a target of *Neurog2* in the embryonic cerebral cortex, is expressed in neuroblasts in the SGZ and might therefore be induced by Neurog2 in the DG lineage (Hodge et al., 2008). However, the function of Neurog2 in DG neurogenesis has not yet been reported.

The T-box TF **Tbr2** is another principal regulator of embryonic neurogenesis, which promotes the generation and proliferation of intermediate progenitors that give rise to pyramidal glutamatergic neurons in the developing cerebral cortex (Englund et al., 2005; Arnold et al., 2008; Sessa et al., 2008). In the developing DG Tbr2 is expressed, like Neurog2, by proliferating progenitor cells in all three matrices (Hodge et al., 2012). Ablation of Tbr2 prevents the generation of IPCs and granule neurons while increasing the proliferation of stem cells in the developing DG, indicating that Tbr2 is necessary for the transition from stem cells to late, differentiating IPCs (Figure 2). Tbr2 has been proposed to exert its functions through direct down-regulation of the stem cell TF Sox2 (Hodge et al., 2012). In addition, Tbr2 is expressed by hem-derived Cajal-Retzius cells and is required for their migration, so defects in the distribution of Cajal-Retzius cells also contribute to the defects in DG morphogenesis in *Tbr2* mutant mice (Hodge et al., 2013).

In the adult brain, Tbr2 is also expressed by IPCs in the two neurogenic regions (Hodge et al., 2008; Roybon et al., 2009). Elimination of Tbr2 from NSCs blocks the production of late IPCs and granule neurons, similar to the phenotype observed in the developing DG. Interestingly, the loss of *Tbr2* also results in an increase in the proliferation of NSCs and their expression of Ascl1 (Hodge et al., 2012). This could be explained by a non-cell autonomous induction of NSC quiescence by IPCs through Notch signaling, as shown in the case of *Prox1* mutant mice (see below), although a more direct role of Tbr2, which is expressed by a small subset of NSCs, is not ruled out (Hodge et al., 2008, 2012).

**Prox1** is a homeobox TF expressed by multiple types of neuronal progenitors and postmitotic cells, including newly born granule cells in the tertiary matrix of the developing DG (Oliver et al., 1993; Li et al., 2009). Prox1 is often used as a marker of the dentate granule neuron lineage, although it is also expressed at low levels in some hippocampal interneurons (Rubin and Kessaris, 2013). Analysis of *Prox1* null mutant mice has shown that *Prox1* is essential during DG development for the proliferation of neuronal progenitors and for the specification of granule cells (Lavado et al., 2010). Remarkably, deleting *Prox1* specifically in post-mitotic granule neurons results in a change in cell identity to that of CA3 pyramidal neurons (Iwano et al., 2012).

In the adult DG, Prox1 is also expressed by late IPCs and its expression is maintained in mature granule neurons (Oliver et al., 1993; Galeeva et al., 2007; Karalay et al., 2011). Conditional deletion of Prox1 in the adult DG impairs the proliferation, survival and differentiation of DG IPCs (Lavado et al., 2010). Even though Prox1 is not expressed in NSCs, the loss of Jagged1-expressing IPCs results in the exhaustion of the NSC pool, due to a decrease in Notch signaling in the stem cells (Lavado et al., 2010).

Both Tbr2 and Prox1 conserve the same roles in the generation of granule cells during embryonic/postnatal hippocampal development and in the adult DG. This suggests that the same genetic programme involving the same key TFs (Neurog2 > Tbr2 > NeuroD1 > Prox1) drives the differentiation of IPCs into glutamatergic cells in the DG from development to adulthood (Hodge et al., 2008, 2012). However this does not hold true for factors acting earlier in the granule cell lineage as discussed in the following section.

## The transition from postnatal to adult neurogenesis: NFIX, Tlx, CcnD2 and Ascl1

Granule neurons in the adult DG are exclusively generated by NSCs located in the SGZ. During embryonic and postnatal development, in contrast, neurons are generated by a heterogeneous population of precursor cells in the dentate matrices (Figures 1, 2). The exact time at which the switch from embryonic to adult modes of neurogenesis occurs in the DG is still not well defined. Several independent pieces of evidence suggest that this happens around the second week of life in mice. At postnatal day 14 (P14), the blades of the DG are already formed and the source of new neurons in the DG becomes restricted to the tertiary matrix, which gradually becomes the SGZ (Pleasure et al., 2000; Sugiyama et al., 2013). At the same time, the first presumptive GFAP- and Nestin-positive NSCs adopt their characteristic location, with the nucleus residing in the SGZ and the basal process extending through the GCL (Li and Pleasure, 2005; Martynoga et al., 2013). The TF NFIX has recently been shown to be required for NSCs to adopt their correct location in the developing DG (Martynoga et al., 2013). It is also around the end of the second postnatal week that specific defects in adult neurogenesis are first noticed in two interesting mouse lines that carry null mutations in the *CcnD2* and *Tlx* genes (Kowalczyk et al., 2004; Shi et al., 2004; Ansorg et al., 2012). In these two mutants, the formation and development of the DG during embryonic and early postnatal life are relatively normal but during late postnatal stages and throughout adulthood, the stem cells fail to maintain their granule neuron production. Similarly, conditional deletion of the proneural gene *Ascl1* results in a complete block of adult neurogenesis whereas it is dispensable for embryonic and early postnatal neurogenesis in the DG (Galichet et al., 2008; Andersen et al., 2014). The adult neurogenesis defects of these mutant mice are discussed in greater detail in the following paragraphs.

Transcription factors of the Nuclear Factor 1 (NFI) family have been implicated in the generation of neuronal and glial cells during hippocampal development. In particular, **NFIX** is expressed at high levels in the DNE, the primordium of the future DG, as early as E14 and *NFIX* null mutant mice present severe defects in DG formation (Campbell et al., 2008; Heng et al., 2014). Neuronal and glial differentiation are delayed in *NFIX* mutants, and these animals present a decrease in the number of Prox1^+^ granule neurons as well as a disorganization of the glial scaffold and a defect in DG morphogenesis (Heng et al., 2014). *NFIX* mutant mice survive until P20, and by that time NSCs are present at a normal density in the DG but are abnormally located, with misplaced cell bodies and misaligned basal processes (Martynoga et al., 2013). The abnormal positioning of *NFIX* mutant NSCs is accompanied by an increase in their proliferation rate, suggesting that they are unable to enter or maintain quiescence. Transcriptome analysis of *NFIX*-inactivated NSCs in the BMP-induced cell culture model of NSC quiescence discussed before, revealed that *NFIX* regulates a large fraction (about one third) of the genes that are regulated between the quiescent and activated NSC states. Interestingly, a significant fraction of *NFIX*-regulated genes control cell adhesion, cell motility or extracellular matrix production (Martynoga et al., 2013). Thus, *NFIX* might be required for NSCs to correctly locate to the SGZ and interact with the different components of the DG niche. In its absence, NSCs may not receive the signals required to remain quiescent. *NFIX* function in adult neurogenesis has not yet been analyzed.

**Tlx**, also known as** Nr2e1**, is an orphan nuclear receptor that is involved in patterning of the embryonic telencephalon. Tlx is expressed throughout the telencephalic VZ, except in the dorso-medial region that will give rise to the hippocampus. Tlx expression remains low in neurogenic regions during late embryonic and postnatal stages and is upregulated only at adult stages (Monaghan et al., 1995; Shi et al., 2004). *Tlx* mutant mice present abnormally small DG and olfactory bulbs, as a result of impaired adult neurogenesis from the SGZ and V-SVZ. The DG of adult *Tlx* mutant mice presents deficits in progenitor proliferation and in the generation of new neurons, which can be reversed by re-expression of *Tlx* in mutant NSCs (Shi et al., 2004; Zhang et al., 2008; Niu et al., 2011; Murai et al., 2014). Overexpression of *Tlx* in the DG of wild-type mice also stimulates neurogenesis and enhances learning and memory performances (Murai et al., 2014). These studies suggest that Tlx promotes the switch of NSCs from quiescence to activation, and several downstream pathways have been implicated in this activity, including the induction of WNT signaling and of *Ascl1* expression and the dowregulation of BMP signaling (Shi et al., 2004; Elmi et al., 2010; Qu et al., 2010; Qin et al., 2014).

**CcnD2** (Cyclin D2) is a key component of the cell cycle machinery that controls the transition between the G1- and S-phases of the cell cycle, together with the other Cyclin D proteins (CcnD1 and CcnD3) and the Cyclin-dependent kinases (CDKs; Sherr, 1994; Ekholm and Reed, 2000). During embryonic development, CcnD1 and CcnD2 promote cell cycle progression but also induce the neuronal differentiation of neural progenitors (Lukaszewicz and Anderson, 2011; Pauklin and Vallier, 2013). Although *CcnD* genes are structurally very similar and can usually substitute for one another functionally, their expression profiles are distinct. As a result, different *CcnD* genes are required for the proliferation and differentiation of distinct progenitor populations, and *CcnD2* has been shown to be specifically required for the proliferation of intermediate precursors in the embryonic cerebral cortex (Komada et al., 2013).

Adult *CcnD2* mutant mice present relatively normal brain morphology, but they are 25% smaller than wild-type mice. As in *Tlx* mutants, *CcnD2* deficient mice present severely reduced rates of neurogenesis in both the V-SVZ and the DG (Kowalczyk et al., 2004). Interestingly, the requirement for *CcnD2* in the proliferation of NSCs builds up progressively from early postnatal stages and becomes absolutely necessary at 4 weeks of age. By then, the DG of *CcnD2* mutant mice is almost completely devoid of proliferating cells and of differentiating neuroblasts, and these defects persist throughout life (Ansorg et al., 2012). *CcnD2* mutant mice have been extensively used to study the contribution of adult-born neurons to memory and behavior. These mice are able to learn spatial tasks known to be dependant on hippocampal functions, but they show reduced flexibility in updating previously learned information, shedding light into the specific functions of new-born cells in the DG (Jaholkowski et al., 2009; Jedynak et al., 2012; Garthe et al., 2014). Strikingly, *CcnD1*, which is expressed during DG development and can promote proliferation and neurogenesis in the adult DG when overexpressed, does not compensate for the loss of *CcnD2* in the adult DG (Shtutman et al., 1999; Tetsu and Mccormick, 1999; Kowalczyk et al., 2004; Klein and Assoian, 2008; Artegiani et al., 2011).

**Ascl1** is a proneural bHLH TF that has crucial roles in the proliferation, neuronal commitment and differentiation of progenitors in the embryonic brain (Bertrand et al., 2002; Castro et al., 2011; Imayoshi and Kageyama, 2014). When overexpressed in astrocytes, fibroblasts and other cell types, it also has the capacity to re-program these cells into neurons (Berninger et al., 2007; Yang et al., 2011; Wapinski et al., 2013). Ascl1 is expressed throughout DG development by progenitor cells in the three matrices, but it is not required for the development of the DG at embryonic stages (Pleasure et al., 2000; Galichet et al., 2008). Moreover, conditional ablation of *Ascl1* during early postnatal stages does not affect stem cell proliferation, suggesting that in its absence, other factors can promote progenitor proliferation in the developing DG (Andersen et al., 2014).

In the adult brain, Ascl1 remains expressed in intermediate progenitors as well as in a subset of actively self-renewing stem cells in the DG and V-SVZ (Pleasure et al., 2000; Parras et al., 2004; Breunig et al., 2007; Kim et al., 2007; Seki et al., 2007; Hodge et al., 2008; Pastrana et al., 2009; Lugert et al., 2012). Strikingly, deletion of *Ascl1* renders adult NSCs permanently quiescent and blocks neurogenesis in both neurogenic niches (Andersen et al., 2014). *Ascl1*-deficient NSCs in the adult DG are not able to respond to neurogenic stimuli, suggesting that Ascl1 is a niche-induced factor that acts at an early step in the switch from quiescence to activation. Although the exact mechanisms by which Ascl1 controls this switch remain to be elucidated, Ascl1 has been shown to directly regulate *CcnD2*, suggesting that one of its roles is to directly promote cell cycle progression of NSCs (Andersen et al., 2014).

Besides its expression in multipotent stem cells and neuronal progenitors, Ascl1 is also expressed in, and required for the specification and differentiation of, a subset of oligodendrocyte precursors in both embryonic and adult brains (Parras et al., 2004; Sugimori et al., 2007; Nakatani et al., 2013). Unexpectedly, overexpression of *Ascl1* in IPCs of the DG promotes their differentiation into oligodendrocytes, a cell type normally not generated by this cell lineage (Jessberger et al., 2008). Why *Ascl1* has such different activities in the adult DG when analyzed by loss-of-function or by gain-of-function approaches is currently unclear.

The analysis of *NFIX, Tlx, CcnD2* and *Ascl1* mouse mutants provides strong evidence that the genetic programmes that promote neurogenesis in the DG during development and in the adult are distinct. However, only null mutants for *NFIX*, *Tlx* and *CcnD2* have been analyzed, and conditional mutant mice will be required to rule out that developmental defects are responsible for the adult hippocampal phenotypes. The similarities between the phenotypes of *Tlx*, *CcnD2*, and *Ascl1* mutants suggest that these genes belong to a common regulatory pathway operating specifically during adult neurogenesis to promote stem cell activation. The severity of their mutant phenotypes demonstrates the fragility of the adult neurogenic process. The loss of one of these genes in adult stem cells cannot be functionally compensated while their loss during DG development has milder or no effects, suggesting that more robust regulatory mechanisms support embryonic and postnatal neurogenesis. The similar or different action during embryonic and adult hippocampal neurogenesis of the molecules and pathways discussed so far is summarized in Table 1.

**Table 1:** **Summary table of the effects during embryonic and adult hippocampal neurogenesis of the main pathways and transcription factors discussed in the text**.

Gene/pathway	Effect during development	Effect in adult neurogenesis	Similar or different?
**Wnt**	Promotes proliferation and neuronal differentiation of neural precursors	Promotes activation of quiescent stem cells and enhances neuronal differentiation	Similar
**BMPR-Ia**	Promotes the proliferation of neural precursors	Maintains stem cells in a quiescent state	Different
**Notch**	Maintains the NSC pool by preventing premature differentiation	Maintains the NSC pool by preventing exit from quiescence	Similar
**Neurog2**	Determines the glutamatergic differentiation of NSCs	Expressed in glutamatergic neuronal precursors but function not directly tested	Similar?
**Tbr2**	Essential for the proliferation and differentiation of IPCs	Essential for the proliferation and differentiation of IPCs	Similar
**Prox1**	Promotes differentiation and determines granule cell identity	Expressed by granule neuron precursor cells, promotes differentiation	Similar
**NFIX**	Required for correct positioning of NSCs in the postnatal DG	Not analyzed (only straight knockout analyzed at P20)	?
**Tlx**	Does not have an important role in development of the DG	Essential for the proliferation of adult NSCs (although only straight knockout tested)	Different
**CcnD2**	Does not have an important role in development of the DG	Essential for the proliferation of adult NSCs (although only straight knockout tested)	Different
**Ascl1**	Does not have an important role in development of the DG	Essential for the proliferation of adult NSCs	Different

## Other signaling pathways: SHH, IGF and neurotransmitters

We will briefly discuss here the roles of other key signaling pathways for which specific roles in hippocampal development or in adult neurogenesis have not been reported.

Sonic Hedgehog signaling has crucial roles in early patterning and cell fate specification in the embryonic brain. Recently, NSCs in the adult DG have been shown to originate from SHH-responsive progenitors in the ventral hippocampus (Li et al., 2013). Sonic Hedgehog signaling has been implicated in the proliferation and maintenance of both DG and V-SVZ adult NSCs (Machold et al., 2003; Álvarez-Buylla and Ihrie, 2014). Although the sources of SHH that regulate V-SVZ and SGZ neurogenesis have not been clearly identified yet, tracing the activity of SHH by the expression of the SHH-inducible gene *Gli1* in *Gli1nLacZ* mice has shown that NSCs in both adult neurogenic regions as well as a fraction of mature astrocytes express the beta galactosidase reporter protein and therefore receive SHH signals (Ahn and Joyner, 2005; Garcia et al., 2010; Ihrie et al., 2011; Petrova et al., 2013). Removal of SHH signaling from V-SVZ stem cells by deletion of the receptor Smoothened has revealed that SHH is necessary for the proliferation and long term maintenance of the stem cells, as well as the subtype specification of the neurons they generate (Palma et al., 2005; Balordi and Fishell, 2007; Kim et al., 2007; Ihrie et al., 2011; Petrova et al., 2013; Merkle et al., 2014). In adult DG stem cells, conditional disruption of primary cilia, which are required for SHH signaling, decreases the production of IPCs, supporting a role for SHH in NSC divisions in the DG as well (Breunig et al., 2008; Amador-Arjona et al., 2011). However, a more direct investigation of the role of SHH in adult DG neurogenesis has not yet been performed.

**Insulin/Insulin Growth Factors (IGFs)** signaling has been shown to stimulate stem cell proliferation and neurogenesis in the adult DG, and this pathway has been proposed to mediate the stimulating effect of physical exercise (running) on neurogenesis (Trejo et al., 2008; Bracko et al., 2012). IGF1, IGF2 and insulin are present in the cerebrospinal fluid but can also reach brain progenitors through the bloodstream (Margolis and Altszuler, 1967; Woods et al., 2003). Insulin/IGF signaling involves both the PI3K/Akt and the MAPK pathways, and the mitogenic activity of IGF2 in DG NSCs requires Akt activity (Bracko et al., 2012). IGF/Akt signaling inactivates the TF FoxO3 through phosphorylation and nuclear exclusion (Calnan and Brunet, 2008; Kenyon, 2010). FoxO3 is expressed by stem cells in the adult DG and V-SVZ and is required to maintain their quiescence (Paik et al., 2009; Renault et al., 2009). Therefore IGF2 might promote stem cell activity in the adult DG in part via Akt-mediated inactivation of the quiescence factor FoxO3. Interestingly, Akt activity has been shown to also increase the stability of the Ascl1 protein in the embryonic brain (Oishi et al., 2009), suggesting that the IGF/Akt might stimulate NSC proliferation by both inactivating a quiescence factor (FoxO3) and stabilizing an activation factor (Ascl1).

Several neurotransmitters have been shown to influence stem cell behavior in the adult V-SVZ and DG, providing a mechanistic link between the activity of particular neuronal populations and the regulation of adult neurogenesis (Berg et al., 2013). The role of the neurotransmitter **GABA** (γ-aminobutyric acid) in the regulation of adult hippocampal neurogenesis has been the focus of several recent studies (Masiulis et al., 2011; Song et al., 2012; Giachino et al., 2014a). GABA can signal through chloride channel-linked GABA_A_ receptors (GABA_A_R) and G protein-coupled GABA_B_ receptors (GABA_B_R), and the loss of both types of receptors increases stem cell proliferation in the DG (Duveau et al., 2011; Song et al., 2012; Giachino et al., 2014a). GABA has well characterized roles in inhibiting proliferation and promoting differentiation of neuronal precursors during embryonic development. In the adult DG, tonic (extra-synaptic) GABA released from parvalbumin-positive interneurons, signals through GABA_A_R to maintain the quiescent state of adult NSCs (Song et al., 2012). In the V-SVZ, GABA_A_ signaling also suppresses stem cell proliferation via a mechanism involving phosphorylation of the histone H2AX (Fernando et al., 2011). **Acetylcholine** has been reported to have an opposite role to GABA and to promote the proliferation of NSCs in the adult hippocampus (Itou et al., 2011). Since physical exercise increases the release of acetylcholine in the hippocampus, this neurotransmitter may contribute to the stimulatory effect of exercise on adult neurogenesis (Suh et al., 2007; Mitsushima et al., 2009; Itou et al., 2011). Other neurotransmitters can affect cell proliferation in the adult V-SVZ or the DG in rodents (Berg et al., 2013), but few studies have assessed their specific effect on stem cells.

## Radial glia and adult NSCs, polarity

The characteristic shape and location of radial glial stem cells in the VZ of the embryonic brain, with their apical side in contact with the brain ventricles and their basal process contacting the pial surface, are vital to their stem cell function and cellular behavior (Götz and Barde, 2005; Götz and Huttner, 2005; Malatesta and Götz, 2013). Stem cells in the adult brain present a similar radial morphology. In the DG, NSCs have an apical side resting in the SGZ and a basal process extending through the GCL and branching into the molecular layer (Fuentealba et al., 2012). The marked polarity of radial glial cells in the embryonic brain has been linked to the reception of distinct signals by different subcellular domains, and to the asymmetric distribution of signal transduction components and fate determinants during cell division (Götz and Huttner, 2005). Although some of these features might be shared by adult NSCs, no study has yet directly examined the interactions of adult NSCs with niche cells *in situ*, the processing of niche signals or the division of adult NSCs. Prominin-1 (CD133), for instance, is segregated to the apical membrane in embryonic radial glia (Weigmann et al., 1997; Kosodo et al., 2004) and has been shown to promote the generation of plasma membrane protrusions (Röper et al., 2000; Corbeil et al., 2001; Kosodo et al., 2004). Prominin-1, in combination with glial markers, has been used to identify and sort NSCs from the embryonic brain and from the V-SVZ and DG in the adult brain (Pinto et al., 2008; Beckervordersandforth et al., 2010, 2014; Walker et al., 2013). Prominin-1 is also apically localized in NSCs of the V-SVZ and SGZ, but its distribution after adult NSC division has not been examined. Similarly, beta-catenin is associated with the apical membrane in embryonic NSCs and is involved in the selection between proliferative and neurogenic modes of division (Machon et al., 2003; Zechner et al., 2003). Despite the importance of beta-catenin in transducing WNT signals, how it is distributed amongst daughter cells after adult NSC divisions is still not known. This also holds true for other features associated with the apical membrane, such as the cilium (and therefore SHH signaling) or with the basal process, such as *CcnD2* mRNA (Tsunekawa et al., 2012). A potential difference between the mechanisms underlying stem cell divisions in the embryonic and adult brain is that during development, daughter cells that retain stem cell properties upon asymmetric division must also retain structures and molecules that promote proliferation, while the opposite might be true for adult NSCs, as their long-term maintenance depends on their ability to return to quiescence. In this respect, differences in the reported preferred angle of division, vertical for embryonic radial glia and horizontal for adult NSCs in the SGZ (Kempermann et al., 2004; Bonaguidi et al., 2011) might be significant. When a DG NSC divides in the adult brain, the basal daughter cell (prospective NSC) might inherit the basal radial process but lose the apical membrane and its associated pro-proliferative signals, which would only persist in the apical daughter cell (prospective IPC) and contribute to its mitotic behavior. Further studies on the polarity of NSCs would greatly contribute to our understanding of the regulation of stem cell divisions in adult neurogenesis.

### Other mechanisms that control neurogenesis

A great variety of additional intrinsic factors and signaling molecules have been shown to regulate adult neurogenesis, but space is lacking to discuss here their function in any detail. Amongst them, the TF **Sox1** is worth mentioning for its neurogenic function during development and its expression in a subset of activated NSCs in the adult DG (Kan et al., 2007; Venere et al., 2012). **Pax6**, expressed by radial glial cells in the developing cerebral cortex, has essential and extensively studied functions in embryonic neurogenesis (Heins et al., 2002). Pax6 is also expressed by NSCs and IPCs in the V-SVZ and the SGZ, and it has a prominent role in adult olfactory neurogenesis, where it is required for the specification of subsets of olfactory bulb interneurons (Hevner et al., 2006; Brill et al., 2008). In neuroblasts from the V-SVZ, Pax6 recruits Brg1, a member of the Brg1/Brm associated factors (BAF) chromatin-remodeling complex. The interaction of Pax6 with the BAF complex is essential for the pro-neurogenic effects of Pax6, and is in particular required to activate transcription of the neurogenic TF genes Sox11, Nfib and Pou3f4 (Ninkovic et al., 2013). However, the role of Pax6 in hippocampal neurogenesis has not yet been studied. The transcriptional repressor **REST**/NRSF (repressor element 1-silencing transcription/neuron-restrictive silencer factor) is highly expressed in stem cells and non-neuronal cell types of the embryo, where it represses the expression of neuronal-specific genes (Chong et al., 1995; Schoenherr and Anderson, 1995; Ballas et al., 2005). In the adult DG, REST is expressed by NSCs and mature granule cells and it is required to maintain NSCs in a quiescent and undifferentiated state, at least in part by direct repression of *Ascl1* and *NeuroD1* (Palm et al., 1998; Calderone et al., 2003; Kuwabara et al., 2004; Jessberger et al., 2007; Gao et al., 2011). MicroRNAs also have major roles in the regulation of adult NSCs (Lopez-Ramirez and Nicoli, 2014). The microRNA miR-124 promotes neuronal differentiation of precursor cells of the V-SVZ lineage, partly through repression of the stem cell factor Sox9 (Cheng et al., 2009). A good example of a microRNA with expression in both embryonic and adult neurogenesis is the brain-enriched microRNA **miR-9**. miR-9 suppresses the expression of key transcriptional regulators of NSCs during development, including Hes1, Tlx and REST (Packer et al., 2008; Zhao et al., 2009; Bonev et al., 2012; Tan et al., 2012; Coolen et al., 2013). Although miR-9 expression has been detected in the adult neurogenic niches in mice, functional studies have not been performed yet (Deo et al., 2006; Kapsimali et al., 2007).

Cell cycle related proteins may also affect specific aspects of adult stem cell behavior, as already discussed for CcnD2. Cyclin-dependent kinases inhibitors such as **p21** and **p27** in the V-SVZ, and p27 and **p57** in the SGZ play vital roles in the maintenance of adult NSC quiescence (Doetsch et al., 2002; Furutachi et al., 2013; Marqués-Torrejón et al., 2013; Andreu et al., 2014). During embryonic neurogenesis, radial glial stem cells have short cell cycles, and the progression from proliferative to differentiative divisions is associated with a lengthening of the cell cycle, specifically of the G1 phase (Lange and Calegari, 2010). Neural stem cells in the adult V-SVZ and SGZ have a shorter cell cycle than early IPCs but this is due to a shorter S-phase rather than to a shorter G1-phase (Brandt et al., 2012; Ponti et al., 2013). Moreover, differentiating neuronal precursors also have a shorter cycle than early IPCs (Brandt et al., 2012), suggesting that the regulation of the cell cycle and the relationships between cell cycle dynamics and differentiation potential may differ between embryonic and adult neurogenesis.

Besides molecular signals known to operate during both developmental and adult neurogenesis, diverse stimuli generated outside of the nervous system can also affect adult neurogenesis. The vasculature, for instance, is a crucial component of the adult neurogenic niche (Shen et al., 2008; Tavazoie et al., 2008). A great variety of endothelial-derived factors (Shen et al., 2004; Ramírez-Castillejo et al., 2006; Kokovay et al., 2010; Gómez-Gaviro et al., 2012; Pineda et al., 2013; Delgado et al., 2014) and recently cell-cell contact between endothelial cells and NSCs (Ottone et al., 2014) have been shown to modulate adult stem cell behaviors, mostly in the context of V-SVZ neurogenesis. Many other stimuli, some of which with no known function in embryonic neurogenesis, such as hormones, inflammation, ageing, or mental disorders are also amongst the many parameters affecting adult neurogenesis that fall beyond the scope of this review (Seki and Arai, 1995; Kuhn et al., 1996; Shingo et al., 2003; Galea et al., 2006; Snyder et al., 2011; Kyritsis et al., 2012; Gebara et al., 2013; Schoenfeld and Cameron, 2014; Sierra et al., 2014; Valero et al., 2014).

## Conclusions

**Figure 3:**
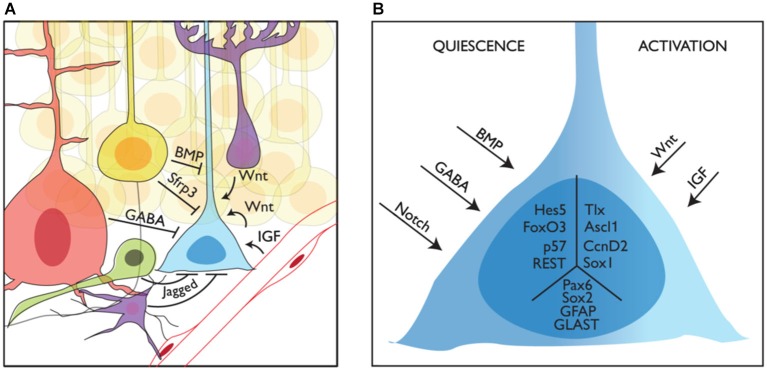
**Niche regulation of mouse adult stem cells in the dentate gyrus. (A)** Representation of a neural stem cell (blue) in the adult subgranular zone of the dentate gyrus and some of its interactions with the niche. Granule neurons (yellow), interneurons (red), intermediate precursors (green) and astrocytes (purple) are shown providing quiescence cues, while blood vessels and astrocytes are shown providing activation cues. **(B)** How quiescence and activation signals are interpreted by adult stem cells is still not known. Here we show several intracellular factors that have been linked to the quiescent (left, Hes5, p. 57, FoxO3 and REST) or active (right, Tlx, Ascl1 and CcnD2) state of stem cells in the adult DG. We also show other factors expressed in NSCs with no clear function in the switch from quiescence to activation (Sox2, Pax6, GFAP and GLAST) in the central part of the schematized cell.

Adult neurogenic niches can be conceptualized as remnants of embryonic signaling centers (i.e., the septum/antihem giving rise to the V-SVZ and the CH generating the SGZ): they are the source of instructive signals that determine the fate of neighboring stem cells. However, in contrast with stem cells in the developing brain that must cope with a continuously changing environment, adult stem cells are surrounded by a relatively stable niche. The V-SVZ and the SGZ niches share many common features. However, while the cellular and molecular composition of the V-SVZ niche has been relatively well investigated, we lack a similar level of understanding of the SGZ niche. Further studies of the signals and cellular interactions that control NSC behavior in the DG will be required before we can appreciate the similarities and divergences in the regulation and function of stem cells in the two adult neurogenic niches (Figure 3).

Genetic analysis of adult neurogenesis suggests that it is an unstable process, since removal of individual regulatory genes often results in dramatic changes in the behavior of adult stem cells. This inherent instability might reflect the strong impact that environmental cues have on stem cell activity. That defects in single quiescence pathways are sufficient to drive the cell cycle re-entry of subsets of stem cells also suggests that different pools of adult stem cells might receive and/or respond to different niche signals. Further investigations will determine whether adult NSCs in the DG are indeed heterogeneous and whether this is due to exposure to different niche signals or to intrinsic differences between distinct NSCs.

## Conflict of interest statement

The authors declare that the research was conducted in the absence of any commercial or financial relationships that could be construed as a potential conflict of interest.

## References

[B1] Ables J. L. Decarolis N. A. Johnson M. A. Rivera P. D. Gao Z. Cooper D. C.. (2010). Notch1 is required for maintenance of the reservoir of adult hippocampal stem cells. J. Neurosci. 30, 10484–10492. 10.1523/JNEUROSCI.4721-09.2010 20685991 PMC2935844

[B2] Ahn S. Joyner A. L. (2005). In vivo analysis of quiescent adult neural stem cells responding to Sonic hedgehog. Nature 437, 894–897. 10.1038/nature03994 16208373

[B3] Altman J. Bayer S. A. (1990). Mosaic organization of the hippocampal neuroepithelium and the multiple germinal sources of dentate granule cells. J. Comp. Neurol. 301, 325–342. 10.1002/cne.903010302 2262594

[B4] Altman J. Das G. D. (1965). Autoradiographic and histological evidence of postnatal hippocampal neurogenesis in rats. J. Comp. Neurol. 124, 319–335. 10.1002/cne.901240303 5861717

[B5] Alunni A. Krecsmarik M. Bosco A. Galant S. Pan L. Moens C. B.. (2013). Notch3 signaling gates cell cycle entry and limits neural stem cell amplification in the adult pallium. Development 140, 3335–3347. 10.1242/dev.095018 23863484 PMC3737716

[B6] Álvarez-Buylla A. Ihrie R. A. (2014). Sonic hedgehog signaling in the postnatal brain. Semin. Cell Dev. Biol. 33, 105–111. 10.1016/j.semcdb.2014.05.008 24862855 PMC4130786

[B7] Amador-Arjona A. Elliott J. Miller A. Ginbey A. Pazour G. J. Enikolopov G.. (2011). Primary cilia regulate proliferation of amplifying progenitors in adult hippocampus: implications for learning and memory. J. Neurosci. 31, 9933–9944. 10.1523/jneurosci.1062-11.2011 21734285 PMC3758574

[B8] Andersen J. Urbán N. Achimastou A. Ito A. Simic M. Ullom K.. (2014). A transcriptional mechanism integrating inputs from extracellular signals to activate hippocampal stem cells. Neuron 83, 1085–1097. 10.1016/j.neuron.2014.08.004 25189209 PMC4157576

[B9] Andreu Z. Khan M. A. González-Gómez P. Negueruela S. Hortigüela R. San Emeterio J.. (2014). The cyclin-dependent kinase inhibitor p27 regulates radial stem cell quiescence and neurogenesis in the adult hippocampus. Stem Cells. [Epub ahead of print]. 10.1002/stem.1832 25185890

[B10] Ansorg A. Witte O. W. Urbach A. (2012). Age-dependent kinetics of dentate gyrus neurogenesis in the absence of cyclin D2. BMC Neurosci. 13:46. 10.1186/1471-2202-13-46 22564330 PMC3403990

[B11] Arnold S. J. Huang G. J. Cheung A. F. Era T. Nishikawa S. Bikoff E. K.. (2008). The T-box transcription factor Eomes/Tbr2 regulates neurogenesis in the cortical subventricular zone. Genes Dev. 22, 2479–2484. 10.1101/gad.475408 18794345 PMC2546697

[B12] Artegiani B. Lindemann D. Calegari F. (2011). Overexpression of cdk4 and cyclinD1 triggers greater expansion of neural stem cells in the adult mouse brain. J. Exp. Med. 208, 937–948. 10.1084/jem.20102167 21482697 PMC3092341

[B13] Ballas N. Grunseich C. Lu D. D. Speh J. C. Mandel G. (2005). REST and its corepressors mediate plasticity of neuronal gene chromatin throughout neurogenesis. Cell 121, 645–657. 10.1016/j.cell.2005.03.013 15907476

[B14] Balordi F. Fishell G. (2007). Hedgehog signaling in the subventricular zone is required for both the maintenance of stem cells and the migration of newborn neurons. J. Neurosci. 27, 5936–5947. 10.1523/jneurosci.1040-07.2007 17537964 PMC6672245

[B15] Basak O. Giachino C. Fiorini E. Macdonald H. R. Taylor V. (2012). Neurogenic subventricular zone stem/progenitor cells are Notch1-dependent in their active but not quiescent state. J. Neurosci. 32, 5654–5666. 10.1523/jneurosci.0455-12.2012 22514327 PMC6703480

[B16] Bayer S. A. (1980a). Development of the hippocampal region in the rat. I. Neurogenesis examined with 3H-thymidine autoradiography. J. Comp. Neurol. 190, 87–114. 10.1002/cne.901900107 7381056

[B17] Bayer S. A. (1980b). Development of the hippocampal region in the rat. II. Morphogenesis during embryonic and early postnatal life. J. Comp. Neurol. 190, 115–134. 10.1002/cne.901900108 7381049

[B18] Beckervordersandforth R. Deshpande A. Schäffner I. Huttner H. B. Lepier A. Lie D. C.. (2014). In vivo targeting of adult neural stem cells in the dentate gyrus by a split-cre approach. Stem Cell Reports 2, 153–162. 10.1016/j.stemcr.2014.01.004 24527389 PMC3923228

[B19] Beckervordersandforth R. Tripathi P. Ninkovic J. Bayam E. Lepier A. Stempfhuber B.. (2010). In vivo fate mapping and expression analysis reveals molecular hallmarks of prospectively isolated adult neural stem cells. Cell Stem Cell 7, 744–758. 10.1016/j.stem.2010.11.017 21112568

[B20] Berg D. A. Belnoue L. Song H. Simon A. (2013). Neurotransmitter-mediated control of neurogenesis in the adult vertebrate brain. Development 140, 2548–2561. 10.1242/dev.088005 23715548 PMC3666382

[B21] Berninger B. Costa M. R. Koch U. Schroeder T. Sutor B. Grothe B.. (2007). Functional properties of neurons derived from in vitro reprogrammed postnatal astroglia. J. Neurosci. 27, 8654–8664. 10.1523/jneurosci.1615-07.2007 17687043 PMC6672931

[B22] Bertrand N. Castro D. S. Guillemot F. (2002). Proneural genes and the specification of neural cell types. Nat. Rev. Neurosci. 3, 517–530. 10.1038/nrn874 12094208

[B23] Blank U. Karlsson G. Karlsson S. (2008). Signaling pathways governing stem-cell fate. Blood 111, 492–503. 10.1182/blood-2007-07-075168 17914027

[B24] Bonaguidi M. A. Mcguire T. Hu M. Kan L. Samanta J. Kessler J. A. (2005). LIF and BMP signaling generate separate and discrete types of GFAP-expressing cells. Development 132, 5503–5514. 10.1242/dev.02166 16314487

[B25] Bonaguidi M. A. Peng C. Y. Mcguire T. Falciglia G. Gobeske K. T. Czeisler C.. (2008). Noggin expands neural stem cells in the adult hippocampus. J. Neurosci. 28, 9194–9204. 10.1523/jneurosci.3314-07.2008 18784300 PMC3651371

[B26] Bonaguidi M. A. Wheeler M. A. Shapiro J. S. Stadel R. P. Sun G. J. Ming G. L.. (2011). In vivo clonal analysis reveals self-renewing and multipotent adult neural stem cell characteristics. Cell 145, 1142–1155. 10.1016/j.cell.2011.05.024 21664664 PMC3124562

[B27] Bond A. M. Peng C. Y. Meyers E. A. Mcguire T. Ewaleifoh O. Kessler J. A. (2014). BMP signaling regulates the tempo of adult hippocampal progenitor maturation at multiple stages of the lineage. Stem Cells 32, 2201–2214. 10.1002/stem.1688 24578327 PMC12952535

[B28] Bonev B. Stanley P. Papalopulu N. (2012). MicroRNA-9 modulates Hes1 ultradian oscillations by forming a double-negative feedback loop. Cell Rep. 2, 10–18. 10.1016/j.celrep.2012.05.017 22840391 PMC4103481

[B29] Bracko O. Singer T. Aigner S. Knobloch M. Winner B. Ray J.. (2012). Gene expression profiling of neural stem cells and their neuronal progeny reveals IGF2 as a regulator of adult hippocampal neurogenesis. J. Neurosci. 32, 3376–3387. 10.1523/jneurosci.4248-11.2012 22399759 PMC3338187

[B30] Brandt M. D. Hübner M. Storch A. (2012). Brief report: adult hippocampal precursor cells shorten S-phase and total cell cycle length during neuronal differentiation. Stem Cells 30, 2843–2847. 10.1002/stem.1244 22987479

[B31] Breunig J. J. Sarkisian M. R. Arellano J. I. Morozov Y. M. Ayoub A. E. Sojitra S.. (2008). Primary cilia regulate hippocampal neurogenesis by mediating sonic hedgehog signaling. Proc. Natl. Acad. Sci. U S A 105, 13127–13132. 10.1073/pnas.0804558105 18728187 PMC2529104

[B32] Breunig J. J. Silbereis J. Vaccarino F. M. Sestan N. Rakic P. (2007). Notch regulates cell fate and dendrite morphology of newborn neurons in the postnatal dentate gyrus. Proc. Natl. Acad. Sci. U S A 104, 20558–20563. 10.1073/pnas.0710156104 18077357 PMC2154470

[B33] Brill M. S. Ninkovic J. Winpenny E. Hodge R. D. Ozen I. Yang R.. (2009). Adult generation of glutamatergic olfactory bulb interneurons. Nat. Neurosci. 12, 1524–1533. 10.1038/nn.2416 19881504 PMC2787799

[B34] Brill M. S. Snapyan M. Wohlfrom H. Ninkovic J. Jawerka M. Mastick G. S.. (2008). A dlx2- and pax6-dependent transcriptional code for periglomerular neuron specification in the adult olfactory bulb. J. Neurosci. 28, 6439–6452. 10.1523/jneurosci.0700-08.2008 18562615 PMC3844782

[B35] Calderone A. Jover T. Noh K. M. Tanaka H. Yokota H. Lin Y.. (2003). Ischemic insults derepress the gene silencer REST in neurons destined to die. J. Neurosci. 23, 2112–2121. 12657670 10.1523/JNEUROSCI.23-06-02112.2003 PMC6741998

[B36] Calnan D. R. Brunet A. (2008). The FoxO code. Oncogene 27, 2276–2288. 10.1038/onc.2008.21 18391970

[B37] Campbell C. E. Piper M. Plachez C. Yeh Y. T. Baizer J. S. Osinski J. M.. (2008). The transcription factor Nfix is essential for normal brain development. BMC Dev. Biol. 8:52. 10.1186/1471-213x-8-52 18477394 PMC2414869

[B38] Caronia G. Wilcoxon J. Feldman P. Grove E. A. (2010). Bone morphogenetic protein signaling in the developing telencephalon controls formation of the hippocampal dentate gyrus and modifies fear-related behavior. J. Neurosci. 30, 6291–6301. 10.1523/jneurosci.0550-10.2010 20445055 PMC2905858

[B39] Castro D. S. Martynoga B. Parras C. Ramesh V. Pacary E. Johnston C.. (2011). A novel function of the proneural factor Ascl1 in progenitor proliferation identified by genome-wide characterization of its targets. Genes Dev. 25, 930–945. 10.1101/gad.627811 21536733 PMC3084027

[B40] Chapouton P. Skupien P. Hesl B. Coolen M. Moore J. C. Madelaine R.. (2010). Notch activity levels control the balance between quiescence and recruitment of adult neural stem cells. J. Neurosci. 30, 7961–7974. 10.1523/jneurosci.6170-09.2010 20534844 PMC6632678

[B41] Chapouton P. Webb K. J. Stigloher C. Alunni A. Adolf B. Hesl B.. (2011). Expression of hairy/enhancer of split genes in neural progenitors and neurogenesis domains of the adult zebrafish brain. J. Comp. Neurol. 519, 1748–1769. 10.1002/cne.22599 21452233

[B42] Cheng X. Hsu C. M. Currle D. S. Hu J. S. Barkovich A. J. Monuki E. S. (2006). Central roles of the roof plate in telencephalic development and holoprosencephaly. J. Neurosci. 26, 7640–7649. 10.1523/jneurosci.0714-06.2006 16855091 PMC6674267

[B43] Cheng L. C. Pastrana E. Tavazoie M. Doetsch F. (2009). miR-124 regulates adult neurogenesis in the subventricular zone stem cell niche. Nat. Neurosci. 12, 399–408. 10.1038/nn.2294 19287386 PMC2766245

[B44] Cheung T. H. Rando T. A. (2013). Molecular regulation of stem cell quiescence. Nat. Rev. Mol. Cell Biol. 14, 329–340. 10.1038/nrm3591 23698583 PMC3808888

[B45] Chong J. A. Tapia-Ramírez J. Kim S. Toledo-Aral J. J. Zheng Y. Boutros M. C.. (1995). REST: a mammalian silencer protein that restricts sodium channel gene expression to neurons. Cell 80, 949–957. 10.1016/0092-8674(95)90298-8 7697725

[B46] Codega P. Silva-Vargas V. Paul A. Maldonado-Soto A. R. Deleo A. M. Pastrana E.. (2014). Prospective identification and purification of quiescent adult neural stem cells from their in vivo niche. Neuron 82, 545–559. 10.1016/j.neuron.2014.02.039 24811379 PMC4360885

[B47] Colak D. Mori T. Brill M. S. Pfeifer A. Falk S. Deng C.. (2008). Adult neurogenesis requires Smad4-mediated bone morphogenic protein signaling in stem cells. J. Neurosci. 28, 434–446. 10.1523/jneurosci.4374-07.2008 18184786 PMC6670509

[B48] Coolen M. Katz S. Bally-Cuif L. (2013). miR-9: a versatile regulator of neurogenesis. Front. Cell Neurosci. 7:220. 10.3389/fncel.2013.00220 24312010 PMC3834235

[B49] Corbeil D. Röper K. Fargeas C. A. Joester A. Huttner W. B. (2001). Prominin: a story of cholesterol, plasma membrane protrusions and human pathology. Traffic 2, 82–91. 10.1034/j.1600-0854.2001.020202.x 11247306

[B50] DeCarolis N. A. Mechanic M. Petrik D. Carlton A. Ables J. L. Malhotra S.. (2013). In vivo contribution of nestin- and GLAST-lineage cells to adult hippocampal neurogenesis. Hippocampus 23, 708–719. 10.1002/hipo.22130 23554226 PMC3732558

[B51] Delgado A. C. Ferrón S. R. Vicente D. Porlan E. Perez-Villalba A. Trujillo C. M.. (2014). Endothelial NT-3 delivered by vasculature and CSF promotes quiescence of subependymal neural stem cells through nitric oxide induction. Neuron 83, 572–585. 10.1016/j.neuron.2014.06.015 25043422

[B52] Del Río J. A. Heimrich B. Borrell V. Förster E. Drakew A. Alcántara S.. (1997). A role for Cajal-Retzius cells and reelin in the development of hippocampal connections. Nature 385, 70–74. 10.1038/385070a0 8985248

[B53] Deng W. Aimone J. B. Gage F. H. (2010). New neurons and new memories: how does adult hippocampal neurogenesis affect learning and memory? Nat. Rev. Neurosci. 11, 339–350. 10.1038/nrn2822 20354534 PMC2886712

[B54] Deo M. Yu J. Y. Chung K. H. Tippens M. Turner D. L. (2006). Detection of mammalian microRNA expression by in situ hybridization with RNA oligonucleotides. Dev. Dyn. 235, 2538–2548. 10.1002/dvdy.20847 16736490

[B55] Doetsch F. Garcia-Verdugo J. M. Alvarez-Buylla A. (1999). Regeneration of a germinal layer in the adult mammalian brain. Proc. Natl. Acad. Sci. U S A 96, 11619–11624. 10.1073/pnas.96.20.11619 10500226 PMC18083

[B56] Doetsch F. Verdugo J. M. Caille I. Alvarez-Buylla A. Chao M. V. Casaccia-Bonnefil P. (2002). Lack of the cell-cycle inhibitor p27Kip1 results in selective increase of transit-amplifying cells for adult neurogenesis. J. Neurosci. 22, 2255–2264. 11896165 10.1523/JNEUROSCI.22-06-02255.2002 PMC6758265

[B57] Dranovsky A. Picchini A. M. Moadel T. Sisti A. C. Yamada A. Kimura S.. (2011). Experience dictates stem cell fate in the adult hippocampus. Neuron 70, 908–923. 10.1016/j.neuron.2011.05.022 21658584 PMC3124009

[B58] Duveau V. Laustela S. Barth L. Gianolini F. Vogt K. E. Keist R.. (2011). Spatiotemporal specificity of GABAA receptor-mediated regulation of adult hippocampal neurogenesis. Eur. J. Neurosci. 34, 362–373. 10.1111/j.1460-9568.2011.07782.x 21722213 PMC3996709

[B59] Ehm O. Göritz C. Covic M. Schäffner I. Schwarz T. J. Karaca E.. (2010). RBPJkappa-dependent signaling is essential for long-term maintenance of neural stem cells in the adult hippocampus. J. Neurosci. 30, 13794–13807. 10.1523/jneurosci.1567-10.2010 20943920 PMC6633732

[B60] Ekholm S. V. Reed S. I. (2000). Regulation of G(1) cyclin-dependent kinases in the mammalian cell cycle. Curr. Opin. Cell Biol. 12, 676–684. 10.1016/s0955-0674(00)00151-4 11063931

[B61] Elmi M. Matsumoto Y. Zeng Z. J. Lakshminarasimhan P. Yang W. Uemura A.. (2010). TLX activates MASH1 for induction of neuronal lineage commitment of adult hippocampal neuroprogenitors. Mol. Cell. Neurosci. 45, 121–131. 10.1016/j.mcn.2010.06.003 20599619

[B62] Encinas J. M. Michurina T. V. Peunova N. Park J. H. Tordo J. Peterson D. A.. (2011). Division-coupled astrocytic differentiation and age-related depletion of neural stem cells in the adult hippocampus. Cell Stem Cell 8, 566–579. 10.1016/j.stem.2011.03.010 21549330 PMC3286186

[B63] Encinas J. M. Sierra A. Valcárcel-Martín R. Martín-Suárez S. (2013). A developmental perspective on adult hippocampal neurogenesis. Int. J. Dev. Neurosci. 31, 640–645. 10.1016/j.ijdevneu.2013.04.001 23588197

[B64] Englund C. Fink A. Lau C. Pham D. Daza R. A. Bulfone A.. (2005). Pax6, Tbr2 and Tbr1 are expressed sequentially by radial glia, intermediate progenitor cells and postmitotic neurons in developing neocortex. J. Neurosci. 25, 247–251. 10.1523/jneurosci.2899-04.2005 15634788 PMC6725189

[B65] Eriksson P. S. Perfilieva E. Björk-Eriksson T. Alborn A. M. Nordborg C. Peterson D. A.. (1998). Neurogenesis in the adult human hippocampus. Nat. Med. 4, 1313–1317. 10.1038/3305 9809557

[B66] Ernst A. Alkass K. Bernard S. Salehpour M. Perl S. Tisdale J.. (2014). Neurogenesis in the striatum of the adult human brain. Cell 156, 1072–1083. 10.1016/j.cell.2014.01.044 24561062

[B67] Faigle R. Song H. (2013). Signaling mechanisms regulating adult neural stem cells and neurogenesis. Biochim. Biophys. Acta 1830, 2435–2448. 10.1016/j.bbagen.2012.09.002 22982587 PMC3541438

[B68] Fan X. Xu H. Cai W. Yang Z. Zhang J. (2003). Spatial and temporal patterns of expression of Noggin and BMP4 in embryonic and postnatal rat hippocampus. Brain Res. Dev. Brain Res. 146, 51–58. 10.1016/j.devbrainres.2003.09.007 14643011

[B69] Fernandes M. Gutin G. Alcorn H. Mcconnell S. K. Hébert J. M. (2007). Mutations in the BMP pathway in mice support the existence of two molecular classes of holoprosencephaly. Development 134, 3789–3794. 10.1242/dev.004325 17913790

[B70] Fernando R. N. Eleuteri B. Abdelhady S. Nussenzweig A. Andäng M. Ernfors P. (2011). Cell cycle restriction by histone H2AX limits proliferation of adult neural stem cells. Proc. Natl. Acad. Sci. U S A 108, 5837–5842. 10.1073/pnas.1014993108 21436033 PMC3078396

[B71] Fuchs E. Tumbar T. Guasch G. (2004). Socializing with the neighbors: stem cells and their niche. Cell 116, 769–778. 10.1016/S0092-8674(04)00255-7 15035980

[B72] Fuentealba L. C. Obernier K. Alvarez-Buylla A. (2012). Adult neural stem cells bridge their niche. Cell Stem Cell 10, 698–708. 10.1016/j.stem.2012.05.012 22704510 PMC3726005

[B73] Furuta Y. Piston D. W. Hogan B. L. (1997). Bone morphogenetic proteins (BMPs) as regulators of dorsal forebrain development. Development 124, 2203–2212. 9187146 10.1242/dev.124.11.2203

[B74] Furutachi S. Matsumoto A. Nakayama K. I. Gotoh Y. (2013). p57 controls adult neural stem cell quiescence and modulates the pace of lifelong neurogenesis. EMBO J. 32, 970–981. 10.1038/emboj.2013.50 23481253 PMC3616292

[B75] Galceran J. Fariñas I. Depew M. J. Clevers H. Grosschedl R. (1999). Wnt3a−/−-like phenotype and limb deficiency in Lef1(−/−)Tcf1(−/−) mice. Genes Dev. 13, 709–717. 10.1101/gad.13.6.709 10090727 PMC316557

[B76] Galea L. A. Spritzer M. D. Barker J. M. Pawluski J. L. (2006). Gonadal hormone modulation of hippocampal neurogenesis in the adult. Hippocampus 16, 225–232. 10.1002/hipo.20154 16411182

[B77] Galeeva A. Treuter E. Tomarev S. Pelto-Huikko M. (2007). A prospero-related homeobox gene Prox-1 is expressed during postnatal brain development as well as in the adult rodent brain. Neuroscience 146, 604–616. 10.1016/j.neuroscience.2007.02.002 17368742

[B78] Galichet C. Guillemot F. Parras C. M. (2008). Neurogenin 2 has an essential role in development of the dentate gyrus. Development 135, 2031–2041. 10.1242/dev.015115 18448566

[B79] Gallo V. Deneen B. (2014). Glial development: the crossroads of regeneration and repair in the CNS. Neuron 83, 283–308. 10.1016/j.neuron.2014.06.010 25033178 PMC4114724

[B80] Gao Z. Ure K. Ding P. Nashaat M. Yuan L. Ma J.. (2011). The master negative regulator REST/NRSF controls adult neurogenesis by restraining the neurogenic program in quiescent stem cells. J. Neurosci. 31, 9772–9786. 10.1523/jneurosci.1604-11.2011 21715642 PMC3365553

[B81] Garcia A. D. Petrova R. Eng L. Joyner A. L. (2010). Sonic hedgehog regulates discrete populations of astrocytes in the adult mouse forebrain. J. Neurosci. 30, 13597–13608. 10.1523/jneurosci.0830-10.2010 20943901 PMC2966838

[B82] Garthe A. Huang Z. Kaczmarek L. Filipkowski R. K. Kempermann G. (2014). Not all water mazes are created equal: cyclin D2 knockout mice with constitutively suppressed adult hippocampal neurogenesis do show specific spatial learning deficits. Genes Brain Behav. 13, 357–364. 10.1111/gbb.12130 24602283 PMC4314690

[B83] Gebara E. Sultan S. Kocher-Braissant J. Toni N. (2013). Adult hippocampal neurogenesis inversely correlates with microglia in conditions of voluntary running and aging. Front. Neurosci. 7:145. 10.3389/fnins.2013.00145 23970848 PMC3747329

[B84] Giachino C. Barz M. Tchorz J. S. Tome M. Gassmann M. Bischofberger J.. (2014a). GABA suppresses neurogenesis in the adult hippocampus through GABAB receptors. Development 141, 83–90. 10.1242/dev.102608 24284211

[B85] Giachino C. Basak O. Lugert S. Knuckles P. Obernier K. Fiorelli R.. (2014b). Molecular diversity subdivides the adult forebrain neural stem cell population. Stem Cells 32, 70–84. 10.1002/stem.1520 23964022 PMC4259462

[B86] Giachino C. Taylor V. (2014). Notching up neural stem cell homogeneity in homeostasis and disease. Front. Neurosci. 8:32. 10.3389/fnins.2014.00032 24611040 PMC3933793

[B87] Goldstein J. Horsley V. (2012). Home sweet home: skin stem cell niches. Cell. Mol. Life Sci. 69, 2573–2582. 10.1007/s00018-012-0943-3 22410738 PMC3449145

[B88] Gómez-Gaviro M. V. Scott C. E. Sesay A. K. Matheu A. Booth S. Galichet C.. (2012). Betacellulin promotes cell proliferation in the neural stem cell niche and stimulates neurogenesis. Proc. Natl. Acad. Sci. U S A 109, 1317–1322. 10.1073/pnas.1016199109 22232668 PMC3268286

[B89] Götz M. Barde Y. A. (2005). Radial glial cells defined and major intermediates between embryonic stem cells and CNS neurons. Neuron 46, 369–372. 10.1016/j.neuron.2005.04.012 15882633

[B90] Götz M. Huttner W. B. (2005). The cell biology of neurogenesis. Nat. Rev. Mol. Cell Biol. 6, 777–788. 10.1038/nrm1739 16314867

[B91] Grandel H. Brand M. (2013). Comparative aspects of adult neural stem cell activity in vertebrates. Dev. Genes Evol. 223, 131–147. 10.1007/s00427-012-0425-5 23179636

[B92] Gross R. E. Mehler M. F. Mabie P. C. Zang Z. Santschi L. Kessler J. A. (1996). Bone morphogenetic proteins promote astroglial lineage commitment by mammalian subventricular zone progenitor cells. Neuron 17, 595–606. 10.1016/s0896-6273(00)80193-2 8893018

[B93] Grove E. A. Tole S. Limon J. Yip L. Ragsdale C. W. (1998). The hem of the embryonic cerebral cortex is defined by the expression of multiple Wnt genes and is compromised in Gli3-deficient mice. Development 125, 2315–2325. 9584130 10.1242/dev.125.12.2315

[B94] Guérout N. Li X. Barnabé-Heider F. (2014). Cell fate control in the developing central nervous system. Exp. Cell Res. 321, 77–83. 10.1016/j.yexcr.2013.10.003 24140262

[B95] Hébert J. M. Mishina Y. Mcconnell S. K. (2002). BMP signaling is required locally to pattern the dorsal telencephalic midline. Neuron 35, 1029–1041. 10.1016/s0896-6273(02)00900-5 12354394

[B96] Heins N. Malatesta P. Cecconi F. Nakafuku M. Tucker K. L. Hack M. A.. (2002). Glial cells generate neurons: the role of the transcription factor Pax6. Nat. Neurosci. 5, 308–315. 10.1038/nn0502-500c 11896398

[B97] Heng Y. H. Mcleay R. C. Harvey T. J. Smith A. G. Barry G. Cato K.. (2014). NFIX regulates neural progenitor cell differentiation during hippocampal morphogenesis. Cereb. Cortex 24, 261–279. 10.1093/cercor/bhs307 23042739 PMC3862270

[B98] Hevner R. F. Hodge R. D. Daza R. A. Englund C. (2006). Transcription factors in glutamatergic neurogenesis: conserved programs in neocortex, cerebellum and adult hippocampus. Neurosci. Res. 55, 223–233. 10.1016/j.neures.2006.03.004 16621079

[B99] Hirabayashi Y. Itoh Y. Tabata H. Nakajima K. Akiyama T. Masuyama N.. (2004). The Wnt/beta-catenin pathway directs neuronal differentiation of cortical neural precursor cells. Development 131, 2791–2801. 10.1242/dev.01165 15142975

[B100] Hodge R. D. Garcia A. J. 3rd Elsen G. E. Nelson B. R. Mussar K. E. Reiner S. L.. (2013). Tbr2 expression in Cajal-Retzius cells and intermediate neuronal progenitors is required for morphogenesis of the dentate gyrus. J. Neurosci. 33, 4165–4180. 10.1523/jneurosci.4185-12.2013 23447624 PMC3623668

[B101] Hodge R. D. Kowalczyk T. D. Wolf S. A. Encinas J. M. Rippey C. Enikolopov G.. (2008). Intermediate progenitors in adult hippocampal neurogenesis: Tbr2 expression and coordinate regulation of neuronal output. J. Neurosci. 28, 3707–3717. 10.1523/jneurosci.4280-07.2008 18385329 PMC6671086

[B102] Hodge R. D. Nelson B. R. Kahoud R. J. Yang R. Mussar K. E. Reiner S. L.. (2012). Tbr2 is essential for hippocampal lineage progression from neural stem cells to intermediate progenitors and neurons. J. Neurosci. 32, 6275–6287. 10.1523/jneurosci.0532-12.2012 22553033 PMC3366485

[B103] Hsieh J. (2012). Orchestrating transcriptional control of adult neurogenesis. Genes Dev. 26, 1010–1021. 10.1101/gad.187336.112 22588716 PMC3360557

[B104] Ihrie R. A. Shah J. K. Harwell C. C. Levine J. H. Guinto C. D. Lezameta M.. (2011). Persistent sonic hedgehog signaling in adult brain determines neural stem cell positional identity. Neuron 71, 250–262. 10.1016/j.neuron.2011.05.018 21791285 PMC3346180

[B105] Imayoshi I. Isomura A. Harima Y. Kawaguchi K. Kori H. Miyachi H.. (2013). Oscillatory control of factors determining multipotency and fate in mouse neural progenitors. Science 342, 1203–1208. 10.1126/science.1242366 24179156

[B106] Imayoshi I. Kageyama R. (2011). The role of Notch signaling in adult neurogenesis. Mol. Neurobiol. 44, 7–12. 10.1007/s12035-011-8186-0 21541768

[B107] Imayoshi I. Kageyama R. (2014). bHLH factors in self-renewal, multipotency and fate choice of neural progenitor cells. Neuron 82, 9–23. 10.1016/j.neuron.2014.03.018 24698265

[B108] Imayoshi I. Sakamoto M. Yamaguchi M. Mori K. Kageyama R. (2010). Essential roles of Notch signaling in maintenance of neural stem cells in developing and adult brains. J. Neurosci. 30, 3489–3498. 10.1523/jneurosci.4987-09.2010 20203209 PMC6634119

[B109] Itou Y. Nochi R. Kuribayashi H. Saito Y. Hisatsune T. (2011). Cholinergic activation of hippocampal neural stem cells in aged dentate gyrus. Hippocampus 21, 446–459. 10.1002/hipo.20761 20054812

[B110] Iwano T. Masuda A. Kiyonari H. Enomoto H. Matsuzaki F. (2012). Prox1 postmitotically defines dentate gyrus cells by specifying granule cell identity over CA3 pyramidal cell fate in the hippocampus. Development 139, 3051–3062. 10.1242/dev.080002 22791897

[B111] Jaholkowski P. Kiryk A. Jedynak P. Ben Abdallah N. M. Knapska E. Kowalczyk A.. (2009). New hippocampal neurons are not obligatory for memory formation; cyclin D2 knockout mice with no adult brain neurogenesis show learning. Learn. Mem. 16, 439–451. 10.1101/lm.1459709 19553382

[B112] Jang M. H. Bonaguidi M. A. Kitabatake Y. Sun J. Song J. Kang E.. (2013). Secreted frizzled-related protein 3 regulates activity-dependent adult hippocampal neurogenesis. Cell Stem Cell 12, 215–223. 10.1016/j.stem.2012.11.021 23395446 PMC3569732

[B113] Jaskelioff M. Muller F. L. Paik J. H. Thomas E. Jiang S. Adams A. C.. (2011). Telomerase reactivation reverses tissue degeneration in aged telomerase-deficient mice. Nature 469, 102–106. 10.1038/nature09603 21113150 PMC3057569

[B114] Jedynak P. Jaholkowski P. Wozniak G. Sandi C. Kaczmarek L. Filipkowski R. K. (2012). Lack of cyclin D2 impairing adult brain neurogenesis alters hippocampal-dependent behavioral tasks without reducing learning ability. Behav. Brain Res. 227, 159–166. 10.1016/j.bbr.2011.11.007 22101301

[B115] Jessberger S. Clark R. E. Broadbent N. J. Clemenson G. D. Jr. Consiglio A. Lie D. C.. (2009). Dentate gyrus-specific knockdown of adult neurogenesis impairs spatial and object recognition memory in adult rats. Learn. Mem. 16, 147–154. 10.1101/lm.1172609 19181621 PMC2661246

[B116] Jessberger S. Nakashima K. Clemenson G. D. Jr. Mejia E. Mathews E. Ure K.. (2007). Epigenetic modulation of seizure-induced neurogenesis and cognitive decline. J. Neurosci. 27, 5967–5975. 10.1523/jneurosci.0110-07.2007 17537967 PMC6672253

[B117] Jessberger S. Toni N. Clemenson G. D. Jr. Ray J. Gage F. H. (2008). Directed differentiation of hippocampal stem/progenitor cells in the adult brain. Nat. Neurosci. 11, 888–893. 10.1038/nn.2148 18587391 PMC2795354

[B118] Kageyama R. Ohtsuka T. Shimojo H. Imayoshi I. (2008). Dynamic Notch signaling in neural progenitor cells and a revised view of lateral inhibition. Nat. Neurosci. 11, 1247–1251. 10.1038/nn.2208 18956012

[B119] Kan L. Jalali A. Zhao L. R. Zhou X. Mcguire T. Kazanis I.. (2007). Dual function of Sox1 in telencephalic progenitor cells. Dev. Biol. 310, 85–98. 10.1016/j.ydbio.2007.07.026 17719572 PMC3437622

[B120] Kapsimali M. Kloosterman W. P. de Bruijn E. Rosa F. Plasterk R. H. Wilson S. W. (2007). MicroRNAs show a wide diversity of expression profiles in the developing and mature central nervous system. Genome Biol. 8, R173. 10.3410/f.1089386.542615 17711588 PMC2375003

[B121] Karalay O. Doberauer K. Vadodaria K. C. Knobloch M. Berti L. Miquelajauregui A.. (2011). Prospero-related homeobox 1 gene (Prox1) is regulated by canonical Wnt signaling and has a stage-specific role in adult hippocampal neurogenesis. Proc. Natl. Acad. Sci. U S A 108, 5807–5812. 10.1073/pnas.1013456108 21436036 PMC3078392

[B122] Katsimpardi L. Litterman N. K. Schein P. A. Miller C. M. Loffredo F. S. Wojtkiewicz G. R.. (2014). Vascular and neurogenic rejuvenation of the aging mouse brain by young systemic factors. Science 344, 630–634. 10.1126/science.1251141 24797482 PMC4123747

[B123] Kempermann G. (2012). New neurons for ’survival of the fittest’. Nat. Rev. Neurosci. 13, 727–736. 10.1038/nrn3319 22948073

[B124] Kempermann G. Jessberger S. Steiner B. Kronenberg G. (2004). Milestones of neuronal development in the adult hippocampus. Trends Neurosci. 27, 447–452. 10.1016/j.tins.2004.05.013 15271491

[B125] Kenyon C. J. (2010). The genetics of ageing. Nature 464, 504–512. 10.1038/nature08980 20336132

[B126] Khalaf-Nazzal R. Francis F. (2013). Hippocampal development - old and new findings. Neuroscience 248C, 225–242. 10.1016/j.neuroscience.2013.05.061 23756184

[B127] Kim E. J. Leung C. T. Reed R. R. Johnson J. E. (2007). In vivo analysis of Ascl1 defined progenitors reveals distinct developmental dynamics during adult neurogenesis and gliogenesis. J. Neurosci. 27, 12764–12774. 10.1523/jneurosci.3178-07.2007 18032648 PMC6673294

[B128] Klein E. A. Assoian R. K. (2008). Transcriptional regulation of the cyclin D1 gene at a glance. J. Cell Sci. 121, 3853–3857. 10.1242/jcs.039131 19020303 PMC4545630

[B129] Kokovay E. Goderie S. Wang Y. Lotz S. Lin G. Sun Y.. (2010). Adult SVZ lineage cells home to and leave the vascular niche via differential responses to SDF1/CXCR4 signaling. Cell Stem Cell 7, 163–173. 10.1016/j.stem.2010.05.019 20682445 PMC2916873

[B130] Komada M. Iguchi T. Takeda T. Ishibashi M. Sato M. (2013). Smoothened controls cyclin D2 expression and regulates the generation of intermediate progenitors in the developing cortex. Neurosci. Lett. 547, 87–91. 10.1016/j.neulet.2013.05.006 23680462

[B131] Kosodo Y. Röper K. Haubensak W. Marzesco A. M. Corbeil D. Huttner W. B. (2004). Asymmetric distribution of the apical plasma membrane during neurogenic divisions of mammalian neuroepithelial cells. EMBO J. 23, 2314–2324. 10.1038/sj.emboj.7600223 15141162 PMC419905

[B132] Kowalczyk A. Filipkowski R. K. Rylski M. Wilczynski G. M. Konopacki F. A. Jaworski J.. (2004). The critical role of cyclin D2 in adult neurogenesis. J. Cell Biol. 167, 209–213. 10.1083/jcb.200404181 15504908 PMC2172537

[B133] Kuang S. Gillespie M. A. Rudnicki M. A. (2008). Niche regulation of muscle satellite cell self-renewal and differentiation. Cell Stem Cell 2, 22–31. 10.1016/j.stem.2007.12.012 18371418

[B134] Kuhn H. G. Dickinson-Anson H. Gage F. H. (1996). Neurogenesis in the dentate gyrus of the adult rat: age-related decrease of neuronal progenitor proliferation. J. Neurosci. 16, 2027–2033. 8604047 10.1523/JNEUROSCI.16-06-02027.1996 PMC6578509

[B135] Kuwabara T. Hsieh J. Muotri A. Yeo G. Warashina M. Lie D. C.. (2009). Wnt-mediated activation of NeuroD1 and retro-elements during adult neurogenesis. Nat. Neurosci. 12, 1097–1105. 10.1038/nn.2360 19701198 PMC2764260

[B136] Kuwabara T. Hsieh J. Nakashima K. Taira K. Gage F. H. (2004). A small modulatory dsRNA specifies the fate of adult neural stem cells. Cell 116, 779–793. 10.1016/s0092-8674(04)00248-x 15035981

[B137] Kyritsis N. Kizil C. Zocher S. Kroehne V. Kaslin J. Freudenreich D.. (2012). Acute inflammation initiates the regenerative response in the adult zebrafish brain. Science 338, 1353–1356. 10.1126/science.1228773 23138980

[B138] Lange C. Calegari F. (2010). Cdks and cyclins link G1 length and differentiation of embryonic, neural and hematopoietic stem cells. Cell Cycle 9, 1893–1900. 10.4161/cc.9.10.11598 20436288

[B139] Lavado A. Lagutin O. V. Chow L. M. Baker S. J. Oliver G. (2010). Prox1 is required for granule cell maturation and intermediate progenitor maintenance during brain neurogenesis. PLoS Biol. 8: e1000460. 10.1371/journal.pbio.1000460 20808958 PMC2923090

[B140] Lavado A. Oliver G. (2014). Jagged1 is necessary for postnatal and adult neurogenesis in the dentate gyrus. Dev. Biol. 388, 11–21. 10.1016/j.ydbio.2014.02.004 24530424 PMC4009513

[B141] Lee S. M. Tole S. Grove E. Mcmahon A. P. (2000). A local Wnt-3a signal is required for development of the mammalian hippocampus. Development 127, 457–467. 10631167 10.1242/dev.127.3.457

[B142] Li G. Fang L. Fernandez G. Pleasure S. J. (2013). The ventral hippocampus is the embryonic origin for adult neural stem cells in the dentate gyrus. Neuron 78, 658–672. 10.1016/j.neuron.2013.03.019 23643936 PMC3669230

[B143] Li G. Kataoka H. Coughlin S. R. Pleasure S. J. (2009). Identification of a transient subpial neurogenic zone in the developing dentate gyrus and its regulation by Cxcl12 and reelin signaling. Development 136, 327–335. 10.1242/dev.025742 19103804 PMC2685973

[B144] Li G. Pleasure S. J. (2005). Morphogenesis of the dentate gyrus: what we are learning from mouse mutants. Dev. Neurosci. 27, 93–99. 10.1159/000085980 16046842

[B145] Lie D. C. Colamarino S. A. Song H. J. Désiré L. Mira H. Consiglio A.. (2005). Wnt signalling regulates adult hippocampal neurogenesis. Nature 437, 1370–1375. 10.1038/nature04108 16251967

[B146] Lim D. A. Tramontin A. D. Trevejo J. M. Herrera D. G. García-Verdugo J. M. Alvarez-Buylla A. (2000). Noggin antagonizes BMP signaling to create a niche for adult neurogenesis. Neuron 28, 713–726. 10.1016/s0896-6273(00)00148-3 11163261

[B147] López-Juárez A. Howard J. Ullom K. Howard L. Grande A. Pardo A.. (2013). Gsx2 controls region-specific activation of neural stem cells and injury-induced neurogenesis in the adult subventricular zone. Genes Dev. 27, 1272–1287. 10.1101/gad.217539.113 23723414 PMC3690400

[B148] Lopez-Ramirez M. A. Nicoli S. (2014). Role of miRNAs and epigenetics in neural stem cell fate determination. Epigenetics 9, 90–100. 10.4161/epi.27536 24342893 PMC3928190

[B149] Lugert S. Basak O. Knuckles P. Haussler U. Fabel K. Götz M.. (2010). Quiescent and active hippocampal neural stem cells with distinct morphologies respond selectively to physiological and pathological stimuli and aging. Cell Stem Cell 6, 445–456. 10.1016/j.stem.2010.03.017 20452319

[B150] Lugert S. Vogt M. Tchorz J. S. Müller M. Giachino C. Taylor V. (2012). Homeostatic neurogenesis in the adult hippocampus does not involve amplification of Ascl1(high) intermediate progenitors. Nat. Commun. 3:670. 10.1038/ncomms1670 22334073

[B151] Lukaszewicz A. I. Anderson D. J. (2011). Cyclin D1 promotes neurogenesis in the developing spinal cord in a cell cycle-independent manner. Proc. Natl. Acad. Sci. U S A 108, 11632–11637. 10.1073/pnas.1106230108 21709239 PMC3136279

[B153] Machold R. Hayashi S. Rutlin M. Muzumdar M. D. Nery S. Corbin J. G.. (2003). Sonic hedgehog is required for progenitor cell maintenance in telencephalic stem cell niches. Neuron 39, 937–950. 10.1016/s0896-6273(03)00593-2 12971894

[B152] Machon O. Backman M. Machonova O. Kozmik Z. Vacik T. Andersen L.. (2007). A dynamic gradient of Wnt signaling controls initiation of neurogenesis in the mammalian cortex and cellular specification in the hippocampus. Dev. Biol. 311, 223–237. 10.1016/j.ydbio.2007.08.038 17916349

[B154] Machon O. van Den Bout C. J. Backman M. Kemler R. Krauss S. (2003). Role of beta-catenin in the developing cortical and hippocampal neuroepithelium. Neuroscience 122, 129–143. 10.1016/s0306-4522(03)00519-0 14596855

[B155] Malatesta P. Götz M. (2013). Radial glia - from boring cables to stem cell stars. Development 140, 483–486. 10.1242/dev.085852 23293279

[B156] Mangale V. S. Hirokawa K. E. Satyaki P. R. Gokulchandran N. Chikbire S. Subramanian L.. (2008). Lhx2 selector activity specifies cortical identity and suppresses hippocampal organizer fate. Science 319, 304–309. 10.1126/science.1151695 18202285 PMC2494603

[B157] Margolis R. U. Altszuler N. (1967). Insulin in the cerebrospinal fluid. Nature 215, 1375–1376. 6055448 10.1038/2151375a0

[B158] Marqués-Torrejón M. A. Porlan E. Banito A. Gómez-Ibarlucea E. Lopez-Contreras A. J. Fernández-Capetillo O.. (2013). Cyclin-dependent kinase inhibitor p21 controls adult neural stem cell expansion by regulating Sox2 gene expression. Cell Stem Cell 12, 88–100. 10.1016/j.stem.2012.12.001 23260487 PMC3714747

[B159] Martynoga B. Mateo J. L. Zhou B. Andersen J. Achimastou A. Urbán N.. (2013). Epigenomic enhancer annotation reveals a key role for NFIX in neural stem cell quiescence. Genes Dev. 27, 1769–1786. 10.1101/gad.216804.113 23964093 PMC3759694

[B160] Masiulis I. Yun S. Eisch A. J. (2011). The interesting interplay between interneurons and adult hippocampal neurogenesis. Mol. Neurobiol. 44, 287–302. 10.1007/s12035-011-8207-z 21956642 PMC3756898

[B161] Merkle F. T. Fuentealba L. C. Sanders T. A. Magno L. Kessaris N. Alvarez-Buylla A. (2014). Adult neural stem cells in distinct microdomains generate previously unknown interneuron types. Nat. Neurosci. 17, 207–214. 10.1038/nn.3610 24362763 PMC4100623

[B162] Merkle F. T. Mirzadeh Z. Alvarez-Buylla A. (2007). Mosaic organization of neural stem cells in the adult brain. Science 317, 381–384. 10.1126/science.1144914 17615304

[B163] Ming G. L. Song H. (2011). Adult neurogenesis in the mammalian brain: significant answers and significant questions. Neuron 70, 687–702. 10.1016/j.neuron.2011.05.001 21609825 PMC3106107

[B164] Mira H. Andreu Z. Suh H. Lie D. C. Jessberger S. Consiglio A.. (2010). Signaling through BMPR-IA regulates quiescence and long-term activity of neural stem cells in the adult hippocampus. Cell Stem Cell 7, 78–89. 10.1016/j.stem.2010.04.016 20621052

[B165] Mirzadeh Z. Merkle F. T. Soriano-Navarro M. Garcia-Verdugo J. M. Alvarez-Buylla A. (2008). Neural stem cells confer unique pinwheel architecture to the ventricular surface in neurogenic regions of the adult brain. Cell Stem Cell 3, 265–278. 10.1016/j.stem.2008.07.004 18786414 PMC2613692

[B166] Mitsushima D. Takase K. Funabashi T. Kimura F. (2009). Gonadal steroids maintain 24 h acetylcholine release in the hippocampus: organizational and activational effects in behaving rats. J. Neurosci. 29, 3808–3815. 10.1523/jneurosci.5301-08.2009 19321777 PMC6665029

[B167] Monaghan A. P. Grau E. Bock D. Schutz G. (1995). The mouse homolog of the orphan nuclear receptor tailless is expressed in the developing forebrain. Development 121, 839–853. 7720587 10.1242/dev.121.3.839

[B168] Morrison S. J. Spradling A. C. (2008). Stem cells and niches: mechanisms that promote stem cell maintenance throughout life. Cell 132, 598–611. 10.1016/j.cell.2008.01.038 18295578 PMC4505728

[B169] Mourikis P. Tajbakhsh S. (2014). Distinct contextual roles for Notch signalling in skeletal muscle stem cells. BMC Dev. Biol. 14:2. 10.1186/1471-213x-14-2 24472470 PMC3903015

[B170] Murai K. Qu Q. Sun G. Ye P. Li W. Asuelime G.. (2014). Nuclear receptor TLX stimulates hippocampal neurogenesis and enhances learning and memory in a transgenic mouse model. Proc. Natl. Acad. Sci. U S A 111, 9115–9120. 10.1073/pnas.1406779111 24927526 PMC4078800

[B171] Nakatani H. Martin E. Hassani H. Clavairoly A. Maire C. L. Viadieu A.. (2013). Ascl1/Mash1 promotes brain oligodendrogenesis during myelination and remyelination. J. Neurosci. 33, 9752–9768. 10.1523/jneurosci.0805-13.2013 23739972 PMC3892435

[B172] Nieto M. Schuurmans C. Britz O. Guillemot F. (2001). Neural bHLH genes control the neuronal versus glial fate decision in cortical progenitors. Neuron 29, 401–413. 10.1016/s0896-6273(01)00214-8 11239431

[B173] Ninkovic J. Steiner-Mezzadri A. Jawerka M. Akinci U. Masserdotti G. Petricca S.. (2013). The BAF complex interacts with Pax6 in adult neural progenitors to establish a neurogenic cross-regulatory transcriptional network. Cell Stem Cell 13, 403–418. 10.1016/j.stem.2013.07.002 23933087 PMC4098720

[B174] Niu W. Zou Y. Shen C. Zhang C. L. (2011). Activation of postnatal neural stem cells requires nuclear receptor TLX. J. Neurosci. 31, 13816–13828. 10.1523/jneurosci.1038-11.2011 21957244 PMC3192402

[B175] Obernier K. Tong C. K. Alvarez-Buylla A. (2014). Restricted nature of adult neural stem cells: re-evaluation of their potential for brain repair. Front. Neurosci. 8:162. 10.3389/fnins.2014.00162 24987325 PMC4060730

[B176] Oishi K. Watatani K. Itoh Y. Okano H. Guillemot F. Nakajima K.. (2009). Selective induction of neocortical GABAergic neurons by the PDK1-Akt pathway through activation of Mash1. Proc. Natl. Acad. Sci. U S A 106, 13064–13069. 10.1073/pnas.0808400106 19549840 PMC2722283

[B177] Okamoto M. Inoue K. Iwamura H. Terashima K. Soya H. Asashima M.. (2011). Reduction in paracrine Wnt3 factors during aging causes impaired adult neurogenesis. FASEB J. 25, 3570–3582. 10.1096/fj.11-184697 21746862

[B178] Oliver G. Sosa-Pineda B. Geisendorf S. Spana E. P. Doe C. Q. Gruss P. (1993). Prox 1, a prospero-related homeobox gene expressed during mouse development. Mech. Dev. 44, 3–16. 10.1016/0925-4773(93)90012-m 7908825

[B179] Orford K. W. Scadden D. T. (2008). Deconstructing stem cell self-renewal: genetic insights into cell-cycle regulation. Nat. Rev. Genet. 9, 115–128. 10.1038/nrg2269 18202695

[B180] Ortiz-Matamoros A. Salcedo-Tello P. Avila-Muñoz E. Zepeda A. Arias C. (2013). Role of wnt signaling in the control of adult hippocampal functioning in health and disease: therapeutic implications. Curr. Neuropharmacol. 11, 465–476. 10.2174/1570159x11311050001 24403870 PMC3763754

[B181] Ottone C. Krusche B. Whitby A. Clements M. Quadrato G. Pitulescu M. E.. (2014). Direct cell-cell contact with the vascular niche maintains quiescent neural stem cells. Nat. Cell Biol. 16, 1045–1056. 10.1038/ncb3045 25283993 PMC4298702

[B182] Packer A. N. Xing Y. Harper S. Q. Jones L. Davidson B. L. (2008). The bifunctional microRNA miR-9/miR-9* regulates REST and CoREST and is downregulated in Huntington’s disease. J. Neurosci. 28, 14341–14346. 10.1523/jneurosci.2390-08.2008 19118166 PMC3124002

[B183] Paik J. H. Ding Z. Narurkar R. Ramkissoon S. Muller F. Kamoun W. S.. (2009). FoxOs cooperatively regulate diverse pathways governing neural stem cell homeostasis. Cell Stem Cell 5, 540–553. 10.1016/j.stem.2009.09.013 19896444 PMC3285492

[B184] Palm K. Belluardo N. Metsis M. Timmusk T. (1998). Neuronal expression of zinc finger transcription factor REST/NRSF/XBR gene. J. Neurosci. 18, 1280–1296. 9454838 10.1523/JNEUROSCI.18-04-01280.1998 PMC6792720

[B185] Palma V. Lim D. A. Dahmane N. Sánchez P. Brionne T. C. Herzberg C. D.. (2005). Sonic hedgehog controls stem cell behavior in the postnatal and adult brain. Development 132, 335–344. 10.1242/dev.01567 15604099 PMC1431583

[B186] Panchision D. M. Pickel J. M. Studer L. Lee S. H. Turner P. A. Hazel T. G.. (2001). Sequential actions of BMP receptors control neural precursor cell production and fate. Genes Dev. 15, 2094–2110. 10.1101/gad.894701 11511541 PMC312756

[B187] Paridaen J. T. Huttner W. B. (2014). Neurogenesis during development of the vertebrate central nervous system. EMBO Rep. 15, 351–364. 10.1002/embr.201438447 24639559 PMC3989667

[B188] Parras C. M. Galli R. Britz O. Soares S. Galichet C. Battiste J.. (2004). Mash1 specifies neurons and oligodendrocytes in the postnatal brain. EMBO J. 23, 4495–4505. 10.1038/sj.emboj.7600447 15496983 PMC526464

[B189] Pastrana E. Cheng L. C. Doetsch F. (2009). Simultaneous prospective purification of adult subventricular zone neural stem cells and their progeny. Proc. Natl. Acad. Sci. U S A 106, 6387–6392. 10.1073/pnas.0810407106 19332781 PMC2669396

[B190] Pauklin S. Vallier L. (2013). The cell-cycle state of stem cells determines cell fate propensity. Cell 155, 135–147. 10.1016/j.cell.2013.08.031 24074866 PMC3898746

[B191] Petrova R. Garcia A. D. Joyner A. L. (2013). Titration of GLI3 repressor activity by sonic hedgehog signaling is critical for maintaining multiple adult neural stem cell and astrocyte functions. J. Neurosci. 33, 17490–17505. 10.1523/JNEUROSCI.2042-13.2013 24174682 PMC3812512

[B192] Pineda J. R. Daynac M. Chicheportiche A. Cebrian-Silla A. Sii Felice K. Garcia-Verdugo J. M.. (2013). Vascular-derived TGF-beta increases in the stem cell niche and perturbs neurogenesis during aging and following irradiation in the adult mouse brain. EMBO Mol. Med. 5, 548–562. 10.1002/emmm.201202197 23526803 PMC3628106

[B193] Pinto L. Mader M. T. Irmler M. Gentilini M. Santoni F. Drechsel D.. (2008). Prospective isolation of functionally distinct radial glial subtypes–lineage and transcriptome analysis. Mol. Cell. Neurosci. 38, 15–42. 10.1016/j.mcn.2008.01.012 18372191

[B194] Pleasure S. J. Collins A. E. Lowenstein D. H. (2000). Unique expression patterns of cell fate molecules delineate sequential stages of dentate gyrus development. J. Neurosci. 20, 6095–6105. 10934259 10.1523/JNEUROSCI.20-16-06095.2000 PMC6772596

[B195] Ponti G. Obernier K. Guinto C. Jose L. Bonfanti L. Alvarez-Buylla A. (2013). Cell cycle and lineage progression of neural progenitors in the ventricular-subventricular zones of adult mice. Proc. Natl. Acad. Sci. U S A 110, E1045–E1054. 10.1073/pnas.1219563110 23431204 PMC3600494

[B196] Qin S. Niu W. Iqbal N. Smith D. K. Zhang C. L. (2014). Orphan nuclear receptor TLX regulates astrogenesis by modulating BMP signaling. Front. Neurosci. 8:74. 10.3389/fnins.2014.00074 24782704 PMC3989729

[B197] Qu Q. Sun G. Li W. Yang S. Ye P. Zhao C.. (2010). Orphan nuclear receptor TLX activates Wnt/beta-catenin signalling to stimulate neural stem cell proliferation and self-renewal. Nat. Cell Biol. 12, 31–40. 10.1038/ncb2001 20010817 PMC2880892

[B198] Ramírez-Castillejo C. Sánchez-Sánchez F. Andreu-Agulló C. Ferrón S. R. Aroca-Aguilar J. D. Sánchez P.. (2006). Pigment epithelium-derived factor is a niche signal for neural stem cell renewal. Nat. Neurosci. 9, 331–339. 10.1038/nn1657 16491078

[B199] Renault V. M. Rafalski V. A. Morgan A. A. Salih D. A. Brett J. O. Webb A. E.. (2009). FoxO3 regulates neural stem cell homeostasis. Cell Stem Cell 5, 527–539. 10.1016/j.stem.2009.09.014 19896443 PMC2775802

[B200] Rickmann M. Amaral D. G. Cowan W. M. (1987). Organization of radial glial cells during the development of the rat dentate gyrus. J. Comp. Neurol. 264, 449–479. 10.1002/cne.902640403 3680638

[B201] Rolando C. Taylor V. (2014). Neural stem cell of the hippocampus: development, physiology regulation and dysfunction in disease. Curr. Top. Dev. Biol. 107, 183–206. 10.1016/b978-0-12-416022-4.00007-x 24439807

[B202] Röper K. Corbeil D. Huttner W. B. (2000). Retention of prominin in microvilli reveals distinct cholesterol-based lipid micro-domains in the apical plasma membrane. Nat. Cell Biol. 2, 582–592. 10.1038/35023524 10980698

[B203] Rowitch D. H. Kriegstein A. R. (2010). Developmental genetics of vertebrate glial-cell specification. Nature 468, 214–222. 10.1038/nature09611 21068830

[B204] Roybon L. Deierborg T. Brundin P. Li J. Y. (2009). Involvement of Ngn2, Tbr and NeuroD proteins during postnatal olfactory bulb neurogenesis. Eur. J. Neurosci. 29, 232–243. 10.1111/j.1460-9568.2008.06595.x 19200230

[B205] Rubin A. N. Kessaris N. (2013). PROX1: a lineage tracer for cortical interneurons originating in the lateral/caudal ganglionic eminence and preoptic area. PLoS One 8:e77339. 10.1371/journal.pone.0077339 24155945 PMC3796451

[B206] Sahay A. Scobie K. N. Hill A. S. O’carroll C. M. Kheirbek M. A. Burghardt N. S.. (2011). Increasing adult hippocampal neurogenesis is sufficient to improve pattern separation. Nature 472, 466–470. 10.1038/nature09817 21460835 PMC3084370

[B207] Schoenfeld T. J. Cameron H. A. (2014). Adult neurogenesis and mental illness. Neuropsychopharmacology [Epub ahead of print]. 10.1038/npp.2014.230 25178407 PMC4262910

[B208] Schoenherr C. J. Anderson D. J. (1995). Silencing is golden: negative regulation in the control of neuronal gene transcription. Curr. Opin. Neurobiol. 5, 566–571. 10.1016/0959-4388(95)80060-3 8580707

[B209] Schuurmans C. Armant O. Nieto M. Stenman J. M. Britz O. Klenin N.. (2004). Sequential phases of cortical specification involve Neurogenin-dependent and -independent pathways. EMBO J. 23, 2892–2902. 10.1038/sj.emboj.7600278 15229646 PMC514942

[B210] Scott I. C. Steiglitz B. M. Clark T. G. Pappano W. N. Greenspan D. S. (2000). Spatiotemporal expression patterns of mammalian chordin during postgastrulation embryogenesis and in postnatal brain. Dev. Dyn. 217, 449–456. 10.1002/(sici)1097-0177(200004)217:4<449::aid-dvdy12>3.0.co;2-8 10767089

[B211] Seib D. R. Corsini N. S. Ellwanger K. Plaas C. Mateos A. Pitzer C.. (2013). Loss of Dickkopf-1 restores neurogenesis in old age and counteracts cognitive decline. Cell Stem Cell 12, 204–214. 10.1016/j.stem.2012.11.010 23395445

[B212] Seki T. Arai Y. (1995). Age-related production of new granule cells in the adult dentate gyrus. Neuroreport 6, 2479–2482. 10.1097/00001756-199512150-00010 8741746

[B213] Seki T. Namba T. Mochizuki H. Onodera M. (2007). Clustering, migration and neurite formation of neural precursor cells in the adult rat hippocampus. J. Comp. Neurol. 502, 275–290. 10.1002/cne.21301 17348003

[B214] Seki T. Sato T. Toda K. Osumi N. Imura T. Shioda S. (2014). Distinctive population of Gfap-expressing neural progenitors arising around the dentate notch migrate and form the granule cell layer in the developing hippocampus. J. Comp. Neurol. 522, 261–283. 10.1002/cne.23508 23983092

[B215] Seo S. Lim J. W. Yellajoshyula D. Chang L. W. Kroll K. L. (2007). Neurogenin and NeuroD direct transcriptional targets and their regulatory enhancers. EMBO J. 26, 5093–5108. 10.1038/sj.emboj.7601923 18007592 PMC2140110

[B216] Seri B. García-Verdugo J. M. Collado-Morente L. Mcewen B. S. Alvarez-Buylla A. (2004). Cell types, lineage and architecture of the germinal zone in the adult dentate gyrus. J. Comp. Neurol. 478, 359–378. 10.1002/cne.20395 15384070

[B217] Sessa A. Mao C. A. Hadjantonakis A. K. Klein W. H. Broccoli V. (2008). Tbr2 directs conversion of radial glia into basal precursors and guides neuronal amplification by indirect neurogenesis in the developing neocortex. Neuron 60, 56–69. 10.1016/j.neuron.2008.09.028 18940588 PMC2887762

[B218] Shen Q. Goderie S. K. Jin L. Karanth N. Sun Y. Abramova N.. (2004). Endothelial cells stimulate self-renewal and expand neurogenesis of neural stem cells. Science 304, 1338–1340. 10.1126/science.1095505 15060285

[B219] Shen Q. Wang Y. Kokovay E. Lin G. Chuang S. M. Goderie S. K.. (2008). Adult SVZ stem cells lie in a vascular niche: a quantitative analysis of niche cell-cell interactions. Cell Stem Cell 3, 289–300. 10.1016/j.stem.2008.07.026 18786416 PMC2747473

[B220] Sherr C. J. (1994). G1 phase progression: cycling on cue. Cell 79, 551–555. 10.1016/0092-8674(94)90540-1 7954821

[B221] Shi Y. Chichung Lie D. Taupin P. Nakashima K. Ray J. Yu R. T.. (2004). Expression and function of orphan nuclear receptor TLX in adult neural stem cells. Nature 427, 78–83. 10.1038/nature02211 14702088

[B222] Shimizu K. Chiba S. Saito T. Kumano K. Hamada Y. Hirai H. (2002). Functional diversity among Notch1, Notch2 and Notch3 receptors. Biochem. Biophys. Res. Commun. 291, 775–779. 10.1006/bbrc.2002.6528 11866432

[B223] Shimojo H. Ohtsuka T. Kageyama R. (2008). Oscillations in notch signaling regulate maintenance of neural progenitors. Neuron 58, 52–64. 10.1016/j.neuron.2008.02.014 18400163

[B224] Shingo T. Gregg C. Enwere E. Fujikawa H. Hassam R. Geary C.. (2003). Pregnancy-stimulated neurogenesis in the adult female forebrain mediated by prolactin. Science 299, 117–120. 10.1126/science.1076647 12511652

[B225] Shtutman M. Zhurinsky J. Simcha I. Albanese C. D’amico M. Pestell R.. (1999). The cyclin D1 gene is a target of the beta-catenin/LEF-1 pathway. Proc. Natl. Acad. Sci. U S A 96, 5522–5527. 10.1073/pnas.96.10.5522 10318916 PMC21892

[B226] Sierra A. Beccari S. Diaz-Aparicio I. Encinas J. M. Comeau S. Tremblay M. E. (2014). Surveillance, phagocytosis and inflammation: how never-resting microglia influence adult hippocampal neurogenesis. Neural Plast. 2014:610343. 10.1155/2014/610343 24772353 PMC3977558

[B227] Sierra A. Encinas J. M. Deudero J. J. Chancey J. H. Enikolopov G. Overstreet-Wadiche L. S.. (2010). Microglia shape adult hippocampal neurogenesis through apoptosis-coupled phagocytosis. Cell Stem Cell 7, 483–495. 10.1016/j.stem.2010.08.014 20887954 PMC4008496

[B228] Simons B. D. Clevers H. (2011). Strategies for homeostatic stem cell self-renewal in adult tissues. Cell 145, 851–862. 10.1016/j.cell.2011.05.033 21663791

[B229] Snyder J. S. Soumier A. Brewer M. Pickel J. Cameron H. A. (2011). Adult hippocampal neurogenesis buffers stress responses and depressive behaviour. Nature 476, 458–461. 10.1038/nature10287 21814201 PMC3162077

[B230] Song J. Zhong C. Bonaguidi M. A. Sun G. J. Hsu D. Gu Y.. (2012). Neuronal circuitry mechanism regulating adult quiescent neural stem-cell fate decision. Nature 489, 150–154. 10.1038/nature11306 22842902 PMC3438284

[B231] Spalding K. L. Bergmann O. Alkass K. Bernard S. Salehpour M. Huttner H. B.. (2013). Dynamics of hippocampal neurogenesis in adult humans. Cell 153, 1219–1227. 10.1016/j.cell.2013.05.002 23746839 PMC4394608

[B232] Stump G. Durrer A. Klein A. L. Lütolf S. Suter U. Taylor V. (2002). Notch1 and its ligands Delta-like and Jagged are expressed and active in distinct cell populations in the postnatal mouse brain. Mech. Dev. 114, 153–159. 10.1016/s0925-4773(02)00043-6 12175503

[B233] Sugimori M. Nagao M. Bertrand N. Parras C. M. Guillemot F. Nakafuku M. (2007). Combinatorial actions of patterning and HLH transcription factors in the spatiotemporal control of neurogenesis and gliogenesis in the developing spinal cord. Development 134, 1617–1629. 10.1242/dev.001255 17344230

[B234] Sugiyama T. Osumi N. Katsuyama Y. (2013). The germinal matrices in the developing dentate gyrus are composed of neuronal progenitors at distinct differentiation stages. Dev. Dyn. 242, 1442–1453. 10.1002/dvdy.24035 24038449

[B235] Suh S. H. Paik I. Y. Jacobs K. (2007). Regulation of blood glucose homeostasis during prolonged exercise. Mol. Cells 23, 272–279. 17646701

[B236] Sun Y. Hu J. Zhou L. Pollard S. M. Smith A. (2011). Interplay between FGF2 and BMP controls the self-renewal, dormancy and differentiation of rat neural stem cells. J. Cell Sci. 124, 1867–1877. 10.1242/jcs.085506 21558414 PMC3096055

[B237] Tan S. L. Ohtsuka T. González A. Kageyama R. (2012). MicroRNA9 regulates neural stem cell differentiation by controlling Hes1 expression dynamics in the developing brain. Genes Cells 17, 952–961. 10.1111/gtc.12009 23134481

[B238] Tavazoie M. Van Der Veken L. Silva-Vargas V. Louissaint M. Colonna L. Zaidi B.. (2008). A specialized vascular niche for adult neural stem cells. Cell Stem Cell 3, 279–288. 10.1016/j.stem.2008.07.025 18786415 PMC6864413

[B239] Taverna E. Götz M. Huttner W. B. (2014). The cell biology of neurogenesis: toward an understanding of the development and evolution of the neocortex. Annu. Rev. Cell Dev. Biol. 30, 465–502. 10.1146/annurev-cellbio-101011-155801 25000993

[B240] Tetsu O. Mccormick F. (1999). Beta-catenin regulates expression of cyclin D1 in colon carcinoma cells. Nature 398, 422–426. 10201372 10.1038/18884

[B241] Trejo J. L. Llorens-Martín M. V. Torres-Alemán I. (2008). The effects of exercise on spatial learning and anxiety-like behavior are mediated by an IGF-I-dependent mechanism related to hippocampal neurogenesis. Mol. Cell. Neurosci. 37, 402–411. 10.1016/j.mcn.2007.10.016 18086533

[B242] Treves A. Tashiro A. Witter M. P. Moser E. I. (2008). What is the mammalian dentate gyrus good for? Neuroscience 154, 1155–1172. 10.1016/j.neuroscience.2008.04.073 18554812

[B243] Tsunekawa Y. Britto J. M. Takahashi M. Polleux F. Tan S. S. Osumi N. (2012). Cyclin D2 in the basal process of neural progenitors is linked to non-equivalent cell fates. EMBO J. 31, 1879–1892. 10.1038/emboj.2012.43 22395070 PMC3343330

[B244] Valero J. Mastrella G. Neiva I. Sánchez S. Malva J. O. (2014). Long-term effects of an acute and systemic administration of LPS on adult neurogenesis and spatial memory. Front. Neurosci. 8:83. 10.3389/fnins.2014.00083 24795557 PMC4001049

[B245] Varela-Nallar L. Inestrosa N. C. (2013). Wnt signaling in the regulation of adult hippocampal neurogenesis. Front. Cell Neurosci. 7:100. 10.3389/fncel.2013.00100 23805076 PMC3693081

[B246] Venere M. Han Y. G. Bell R. Song J. S. Alvarez-Buylla A. Blelloch R. (2012). Sox1 marks an activated neural stem/progenitor cell in the hippocampus. Development 139, 3938–3949. 10.1242/dev.081133 22992951 PMC3472585

[B247] Walker T. L. Wierick A. Sykes A. M. Waldau B. Corbeil D. Carmeliet P.. (2013). Prominin-1 allows prospective isolation of neural stem cells from the adult murine hippocampus. J. Neurosci. 33, 3010–3024. 10.1523/jneurosci.3363-12.2013 23407958 PMC6619213

[B248] Wapinski O. L. Vierbuchen T. Qu K. Lee Q. Y. Chanda S. Fuentes D. R.. (2013). Hierarchical mechanisms for direct reprogramming of fibroblasts to neurons. Cell 155, 621–635. 10.1016/j.cell.2013.09.028 24243019 PMC3871197

[B249] Weigmann A. Corbeil D. Hellwig A. Huttner W. B. (1997). Prominin, a novel microvilli-specific polytopic membrane protein of the apical surface of epithelial cells, is targeted to plasmalemmal protrusions of non-epithelial cells. Proc. Natl. Acad. Sci. U S A 94, 12425–12430. 10.1073/pnas.94.23.12425 9356465 PMC24979

[B250] Wilkinson G. Dennis D. Schuurmans C. (2013). Proneural genes in neocortical development. Neuroscience 253, 256–273. 10.1016/j.neuroscience.2013.08.029 23999125

[B251] Woods S. C. Seeley R. J. Baskin D. G. Schwartz M. W. (2003). Insulin and the blood-brain barrier. Curr. Pharm. Des. 9, 795–800. 10.2174/1381612033455323 12678878

[B252] Yang N. Ng Y. H. Pang Z. P. Südhof T. C. Wernig M. (2011). Induced neuronal cells: how to make and define a neuron. Cell Stem Cell 9, 517–525. 10.1016/j.stem.2011.11.015 22136927 PMC4377331

[B253] Yoshida M. Assimacopoulos S. Jones K. R. Grove E. A. (2006). Massive loss of Cajal-Retzius cells does not disrupt neocortical layer order. Development 133, 537–545. 10.1242/dev.02209 16410414

[B254] Zechner D. Fujita Y. Hülsken J. Müller T. Walther I. Taketo M. M.. (2003). beta-Catenin signals regulate cell growth and the balance between progenitor cell expansion and differentiation in the nervous system. Dev. Biol. 258, 406–418. 10.1016/s0012-1606(03)00123-4 12798297

[B255] Zhang L. Yang X. Yang S. Zhang J. (2011). The Wnt /beta-catenin signaling pathway in the adult neurogenesis. Eur. J. Neurosci. 33, 1–8. 10.1111/j.1460-9568.2010.7483.x 21073552

[B256] Zhang C. L. Zou Y. He W. Gage F. H. Evans R. M. (2008). A role for adult TLX-positive neural stem cells in learning and behaviour. Nature 451, 1004–1007. 10.1038/nature06562 18235445

[B257] Zhao C. Deng W. Gage F. H. (2008). Mechanisms and functional implications of adult neurogenesis. Cell 132, 645–660. 10.1016/j.cell.2008.01.033 18295581

[B258] Zhao C. Sun G. Li S. Shi Y. (2009). A feedback regulatory loop involving microRNA-9 and nuclear receptor TLX in neural stem cell fate determination. Nat. Struct. Mol. Biol. 16, 365–371. 10.1038/nsmb.1576 19330006 PMC2667220

[B259] Zhou C. J. Zhao C. Pleasure S. J. (2004). Wnt signaling mutants have decreased dentate granule cell production and radial glial scaffolding abnormalities. J. Neurosci. 24, 121–126. 10.1523/jneurosci.4071-03.2004 14715945 PMC6729560

