# Erratum on: Neurogenesis in the embryonic and adult brain: same regulators, different roles

**DOI:** 10.3389/fncel.2015.00160

**Published:** 2015-04-23

**Authors:** Frontiers Production Office

**Affiliations:** Frontiers Production Office, FrontiersSwitzerland

**Keywords:** hippocampal neurogenesis, development of the hippocampus, regulation of adult neurogenesis, neural stem cell quiescence, niche signals in adult neurogenesis

Reason for Erratum:

Due to a typesetting error the spelling of a few terms was incorrect in “Notch Signaling” Section, paragraph 4. This error does not change the scientific conclusions of the article in any way. The publisher apologizes for this error and the correct text appears below:

A tantalizing hypothesis for the mechanism underlying Notch function in stem cell quiescence comes from embryonic data showing that the levels of Hes proteins and proneural bHLHs oscillate in neural precursor cells (Imayoshi et al., 2013). Hes proteins are bHLH TFs that are induced by Notch activity and act as potent repressors of gene expression, and proneural bHLH genes are amongst their main targets (Imayoshi and Kageyama, 2014). Hes transcripts and proteins oscillate with a frequency of 2–3 h, because Hes proteins repress their own transcription and because this repression is only transient due to their short half-lives (Shimojo et al., 2008; Imayoshi et al., 2013). The oscillation of Hes proteins drives in opposite phase the oscillation of their targets, including the proneural proteins Neurog2 and Ascl1 (Shimojo et al., 2008; Imayoshi et al., 2013). Ascl1 has been shown to have two opposing roles in embryonic neurogenesis, promoting progenitor proliferation and driving their cell cycle exit and differentiation (Castro et al., 2011). Interestingly, the oscillating expression of Ascl1 promotes the proliferation of neural progenitors, while its stable expression instead drives differentiation (Imayoshi et al., 2013). The mechanisms that convert different Ascl1 dynamics into the activation of different gene expression programmes, promoting proliferation and differentiation, respectively, remain unknown. Whether Hes and Ascl1 proteins also oscillate in adult progenitors has not yet been established. Adult NSCs express high levels of Hes proteins, but an initial reduction in the amount of Notch signaling might initiate their oscillatory expression, which would thus trigger the oscillation of Ascl1 expression and the proliferation of NSCs. Subsequently, a complete loss of Notch activity in IPCs would stabilize Ascl1 expression and promote neuronal differentiation. Several observations support such a scenario. Ascl1 expression is indeed increased upon loss of RPBJk in NSCs, showing that also in the adult DG, Notch signaling suppresses its expression (Andersen et al., 2014). Differences in the intensity of Notch signaling have been singled out as possible causes of the heterogeneity in adult NSC behavior (Giachino and Taylor, 2014). Moreover, Ascl1 expression in adult NSCs is excluded from quiescent NSCs and confined to about a third of activated NSCs, suggesting that Ascl1 has indeed a dynamic expression in proliferating NSCs (Andersen et al., 2014). Further analysis will determine the importance of the interactions between the Notch-Hes pathways and Ascl1 in regulating the transitions between quiescent and activated NSCs and between NSCs and IPCs.

The original article has been updated.
